# Genotype-phenotype associations in French patients with phenylketonuria and importance of genotype for full assessment of tetrahydrobiopterin responsiveness

**DOI:** 10.1186/s13023-015-0375-x

**Published:** 2015-12-15

**Authors:** Elise Jeannesson-Thivisol, François Feillet, Céline Chéry, Pascal Perrin, Shyue-Fang Battaglia-Hsu, Bernard Herbeth, Aline Cano, Magalie Barth, Alain Fouilhoux, Karine Mention, François Labarthe, Jean-Baptiste Arnoux, François Maillot, Catherine Lenaerts, Cécile Dumesnil, Kathy Wagner, Daniel Terral, Pierre Broué, Loïc de Parscau, Claire Gay, Alice Kuster, Antoine Bédu, Gérard Besson, Delphine Lamireau, Sylvie Odent, Alice Masurel, Jean-Louis Guéant, Fares Namour

**Affiliations:** 1 Reference Center for Inherited Metabolic Diseases, University Hospital of Nancy, 9 ave Forêt de Haye, BP 184, 54511 Vandoeuvre-lès-Nancy, France; 2 INSERM U954, Department of Nutrition-Genetics-Environmental Risk Exposure, University of Lorraine, 9 ave Forêt de Haye, BP 184, 54511 Vandoeuvre-lès-Nancy, France; 3 Reference Center for Inherited Metabolic Diseases, Timone Hospital, Marseille, France; 4 Department of Biochemistry and Genetics, Angers University Hospital, Angers, France; 5 Reference Center for Inherited Metabolic Diseases, Hospices Civils de Lyon, Bron, France; 6 Reference Center for Inherited Metabolic Diseases, Jeanne de Flandres Hospital, Lille, France; 7 Department of Pediatric Medicine, Clocheville Hospital, Tours, France; 8 Reference Center for Inherited Metabolic Diseases, Necker-Enfants Malades Hospital, Paris, France; 9 Department of Internal Medicine, Tours University Hospital, Tours, France; 10 Department of Pediatrics, Amiens University-Hospital, Amiens, France; 11 Pediatric Hematology and Oncology, Rouen University-Hospital, Rouen, France; 12 Department of Pediatrics, Lenval Hospital, Nice, France; 13 Department of Pediatrics, Hotel-Dieu Hospital, Clermont-Ferrand, France; 14 Department of Pediatric Hepatology and Metabolic Diseases, Children Hospital, Toulouse, France; 15 Department of Pediatrics, CHRU Morvan, Brest, France; 16 Department of Pediatrics, Saint-Etienne University-Hospital, Saint-Etienne, France; 17 Pediatric Department, Nantes University Hospital, Nantes, France; 18 Neonatology Department, Mère-Enfant Hospital, Limoges, France; 19 Department of Neurology, University Hospital of Grenoble, Grenoble, France; 20 Department of Pediatrics, Pellegrin-Enfants Hospital, Bordeaux, France; 21 Department of Clinical Genetics, Rennes University Hospital, Rennes, France; 22 Department of Medical Genetics, Dijon University-Hospital, Dijon, France

**Keywords:** Phenylketonuria, Tetrahydrobiopterin, BH4-loading test, Genotype, BH4-responsiveness

## Abstract

**Background:**

Mutations in Phenylalanine Hydroxylase (*PAH*) gene cause phenylketonuria. Sapropterin (BH4), the enzyme cofactor, is an important therapeutical strategy in phenylketonuria. However, *PAH* is a highly polymorphic gene and it is difficult to identify BH4-responsive genotypes. We seek here to improve prediction of BH4-responsiveness through comparison of genotypes, BH4-loading test, predictions of responsiveness according to the literature and types and locations of mutations.

**Methods:**

A total of 364 French patients among which, 9 % had mild hyperphenylalaninemia, 17.7 % mild phenylketonuria and 73.1 % classical phenylketonuria, benefited from a 24-hour BH4-loading test and had the *PAH* gene sequenced and analyzed by Multiplex Ligation Probe Amplification.

**Results:**

Overall, 31.6 % of patients were BH4-responsive. The number of different mutations found was 127, including 26 new mutations. The mutations c.434A > T, c.500A > T, c.529G > C, c.1045 T > G and c.1196 T > C were newly classified as being BH4-responsive. We identified 261 genotypes, among which 46 were newly recognized as being BH4-responsive. Even though patients carry 2 responsive alleles, BH4-responsiveness cannot be predicted with certainty unless they present mild hyperphenylalaninemia. BH4-responsiveness cannot be predicted in patients carrying one responsive mutation only. In general, the milder the phenotype is, the stronger the BH4-response is. Almost exclusively missense mutations, particularly in exons 12, 11 and 8, are associated with BH4-responsiveness and any other type of mutation predicts a negative response.

**Conclusions:**

This study is the first of its kind, in a French population, to identify the phenotype associated with several combinations of *PAH* mutations. As others, it highlights the necessity of performing simultaneously BH4 loading test and molecular analysis in monitoring phenylketonuria patients.

**Electronic supplementary material:**

The online version of this article (doi:10.1186/s13023-015-0375-x) contains supplementary material, which is available to authorized users.

## Background

Phenylketonuria (PKU, OMIM 261600) is an autosomal recessive disorder caused by a deficient hepatic phenylalanine hydroxylase (PAH) activity [1]. To date, no less than 890 disease causing mutations have been registered to the BioPKU database [2]. In general, the genotype dictates the phenotype [3]. Individuals with simultaneous presence of two null alleles exhibit the classic PKU (cPKU) phenotype, whereas individuals with mutations bearing residual enzyme activity display the mild PKU (mPKU) or the mild hyperphenylalaninemia (mHP).

PAH is a homotetrameric or homodimeric enzyme, whose activity is regulated by the binding of its substrate, Phenylalanine (Phe), and its cofactor, tetrahydrobiopterin (BH4). Each subunit of this multimeric enzyme is composed of the regulatory, the catalytic and the oligomerization domains [4].

The mainstay of the treatment for PKU remains the low-phenylalanine diet [5]. Additional strategy involving the use of sapropterin, a specific pharmacological chaperone similar to the physiologic cofactor tetrahydrobiopterin, is being introduced [6]. Sapropterin enhances PAH enzymatic activity, through multiple mechanisms including (1) stabilization of PAH dimer/tetramer, (2) up-regulation of gene expression, and (3) stabilization of mRNA [4, 7, 8]. However, sapropterin is not efficient in all patients. The degree of sapropterin-responsiveness depends on the presence of responsive mutations [9] having still residual PAH activity [10]. Since PAH is a multimeric protein, it depends also on the allelic complementation of the particular genotype [11, 12].

Numerous studies have investigated mutations in the *PAH* gene as well as prediction of BH4-responsiveness based on genotypes. However, only few have additionally performed BH4-loading tests [13–20]. Thus the status of several genotypes is unknown and whether they are BH4-responsive or not needs to be clarified.

We have performed a systematical research of mutations in French PKU patients and systematically evaluated the response to oral BH4-loading test. The aim was to improve the prediction of BH4-responsiveness by relating genetic information such as type, location and combination of mutations, to BH4-loading test responses.

## Methods

### Patients

A total of 364 hyperphenylalaninemic patients, including 42 siblings, were investigated. They represented mixed origin people from 20 French Centers for Inborn Errors of Metabolism. According to their blood Phenylalanine concentration at time of diagnosis, these patients were assigned to one of the three phenotype categories: mild hyperphenylalaninemia (mHP, 180 < Phe < 600 μmol/L); mild phenylketonuria (mPKU, 600 < Phe < 1200 μmol/L) and classical phenylketonuria (cPKU, Phe > 1200 μmol/L) [21].

### BH4-responsiveness

The BH4 oral loading test protocol has been standardized and published in the French High Health Authority (HAS) 2010 recommendations [22]. Patients were considered as BH4-responsive if an oral bolus of BH4 dose (20 mg/kg body weight) lowered blood Phe concentration by at least 30 % at one time point within 24 hours. In 35 % of patients, BH4-loading test was performed during the neonatal period, in 42 % between 1 month and 18 years and in 23 % during adulthood.

### DNA isolation, PCR amplification, dHPLC (denatured High Performance Liquid Chromatography) and sequencing

Informed consent for genotype assessment was obtained from patients (or parents) for being included in the study. Genotyping analyses were conducted in a single laboratory. Genomic DNA was extracted from blood samples using the Nucleon BACC3 Genomic DNA Extraction Kit (GE Healthcare). PCR was carried out with 13 sets of oligonucleotides amplifying all 13 exons of the gene and the exon-intron junctions. Amplicons were separated by dHPLC as previously reported [23]. Fragments showing an abnormal dHPLC pattern were purified (Qiaquick PCR purification kit, Qiagen), quantified and sequenced using the ABI PRISM Big Dye terminator V1.1 sequencing kit and the 3100-Avant Genetic Analyzer (Applied Biosystems). When no mutation was identified with dHPLC, all exons were sequenced.

### Multiplex Ligation-dependent Probe Amplification (MLPA) analysis

When only one mutation was found, MLPA analysis was performed with the SALSA MLPA kit P055 (MRC) in order to detect large deletions or duplications.

### Variants classification

A mutation was considered as novel if not listed in the NCBI database [24] and hence was submitted to this database. An unclassified mutation was considered as BH4-responsive when BH4 challenging was positive and the genotype was either homozygous for this mutation or compound heterozygous combining this mutation with a null mutation [10, 12]. A null mutation is a nonsense mutation or a mutation yielding a protein with less than 1 % predicted residual activity (PRA). If a homozygous genotype was found to be associated with unresponsiveness in at least five patients, the mutated allele was considered as non-responsive. In addition, a genotype was newly recognized as responsive if 1) there was at least one responsive patient in the present study and 2) it was not previously described at the BioPKU database. All genotypes have been submitted to the BioPKU database [2].

### Molecular modeling

Missense mutations newly described as BH4-responsive were modeled by *in silico* analysis using Pymol molecular visualization system and the published structure of truncated PAH with Protein Data Bank entry 1MMK (catalytic domain and dimerization motif).

Secondary structure was drawn with Dog 2.0 software using information from Erlandsen and colleagues [25], UniprotKB/Swissprot (P00439) and NCBI Reference sequence NP_000268.1.

### Statistical analysis

Statistical analysis was performed by using the SAS software package version 9.3 (SAS Institute). Genotypes, mutations and related descriptive characteristics were presented as frequency, mean and/or percentage. Associations were tested using *t*-test, Chi-square (Chi-2) or Fisher's exact tests. Tests were judged significant at p ≤ 0.05.

## Results

### Phenotype distribution

Thirty-three patients were identified as mHP (9 %), 65 as mPKU (17.7 %) and 266 as cPKU (73.1 %). Patients with siblings similarly affected by PKU appeared to share the same phenotype. 24 hours after BH4-loading approximately one third of patients (31.6 %) experienced a reduction in their Phe concentration of at least 30 %. Overall, more responders were found in groups with the less severe phenotypes, with the positive response rate at 90.9 % for mHP, 69.2 % for mPKU and 14.7 % for cPKU, respectively (*p* < 0.0001; Fisher exact test) (Additional file 1: Figure S1). In addition, the milder the phenotype is, the higher the response rate is. Indeed, 71 % of mHP with a positive test versus 37 % of cPKU with a positive test presented with a responsive rate higher than 50 %.

mHP patients BH4 responders were tested at initial Phe concentrations at the start of BH4-testing (H0) ranking between 114 and 990 μmol/L (mean = 366). Three patients among 33 with mHP were BH4 non-responsive (c.[1045 T > C]; [1243G > A] (p. [Ser349Pro]; [Asp415Asn]); c. [734 T > C]; [1315 + 1G > A] (p. [Val245Ala]; [?]) and c. [261C > A]; [1065 + 1G > A] (p. [Ser87Arg]; [?]) genotypes). Two of them were tested between 3 and 18 yo, both at initial Phe concentration of 144 μmol/L, lower that minimum concentration required for testing. For PKU, there was no difference in Phe concentration at the start of BH4 testing between responsive aPKU (*n* = 37; mH0 = 600 μmol/L (216–1116)) and non-responsive aPKU (*n* = 15; mH0 = 672 μmol/L (318–1026)) neither between responsive cPKU (*n* = 29; mH0 = 1206 μmol/L (342–2160)) and non-responsive cPKU (*n* = 170; mH0 = 1374 μmol/L (300–4320)) (*T*-test; p values = 0.28 and 0.22 respectively).

### Mutation detection rate and mutations spectrum

No mutation was found for one classical PKU patient and only a single heterozygous pathogenic mutation could be identified in one mHP, 2 mPKU and 2 cPKU. Twelve deletions and one duplication were found by MLPA. Mutation detection rate was 97.3 % with sequencing alone and 99.05 % when combined with MLPA.

A total of 127 different mutations were detected among which 26 new ones. Mutations were mapped to the catalytic (63.8 %), regulatory (17.3 %) and oligomerization (2.4 %) domains and 15.7 % localized in intronic regions. One deletion spanned across catalytic and oligomerization domains. Mutations types were as follows: 59.8 % missense, 15.7 % splice-site, 9.4 % frameshift small deletions or insertions, 8.7 % nonsense mutations, as well as 3.9 % large deletions, 1.6 % in-frame small deletions or duplications and 0.8 % large duplications.

Sixty-one mutations were private mutations. The most frequent mutation found was c.1066-11G > A (8.6 %). Some of the most frequent alleles (frequency >1 %) were significantly associated with the severity of the clinical phenotype like c.1066-11G > A (PRA 0) and c.1055delG (p.Gly352Valfs*48) (Fisher exact test, *p* < 0.05). On the other hand, c.1241A > G (p.Tyr414Cys), c.1169A > G (p. Glu390Gly), c.1208C > T (p.Ala403Val), c.204A > T (p.Arg68Ser) and c.898G > T (p.Ala300Ser), known to have PRA >30 %, were associated with moderate phenotypes (mHP and mPKU, Fisher exact test, *p* < 0.001). Although predicted residual activity of c.782G > A (p.Arg261Gln) is 38.5, this mutation was found to be more frequent in the cPKU group (Fisher exact test, *p* < 0.05).

### Mutations responsiveness to BH4

Data available in the literature indicated that among the 127 pathogenic mutations we found, 28 are BH4-responsive and 17 BH4-unresponsive. Among the 82 left mutations, we succeeded in classifying 5 as BH4-responsive and 7 as BH4-unresponsive while the 70 others remained with an unknown status. The new mutation c.1196 T > C (p.Val399Ala) was considered BH4-responsive since after BH4-loading test (initial Phe concentration 1020 μmol/L), a 97 % decrease in Phe concentration was observed in a compound heterozygous patient carrying this mutation with c.1066-11G > A mutation generating a protein with negligible residual activity. Similar conclusion was drawn for the c.1045 T > G mutation (p.Ser349Ala) since positive BH4-responses were observed in one homozygous mPKU patient and another compound heterozygous patient carrying an additional non-responsive allele. The mutations c.434A > T (p.Asp145Val), c.500A > T (p.Asn167Ile) and c.529G > C (p.Val177Leu) were also classified as BH4-responsive since they were found in compound heterozygous patients carrying a second null mutation. These mutations were mapped to the catalytic domain of the protein and are shown on a modeled tridimensional structure representing the PAH protein complexed with BH4, thienylalanine (analog substrate) and iron (Additional file 2: Figure S2 and Additional file 3: Figure S3). Another group of five unclassified mutations fitted the criteria to be classified as responsive mutations: c.266_267insC (p.Ala90Cfs*12), c.1249 T > A (p.Tyr417Asn), c.887A > G (p.Asp296Gly newly described mutation), c.1199 + 1G > C and c.493G > A (p.Ala165Thr) but where classified as unclear especially because they were observed in patients with a BH4-responsive rate comprised between 30 and 40 percent (see Discussion). Thus c.[266_267insC]; [842C > T] genotype was observed in one patient displaying a 36 % decrease in Phe concentration (H0 1560 μmol/L; tested between 3 and 10 yo) and c. [1249 T > A]; [1315 + 1G > A] genotype was observed in one mPKU with a 37 % decrease in Phe concentration (H0 702 μmol/L; neonatal test). The c.887A > G (p.Asp296Gly) mutation was found in one patient combined with the non-responsive c.1222C > T mutation (p. Arg408Trp). This patient showed a 36 % decrease in Phe concentration (H0 1326 μmol/L; tested between 10 and 18 yo), however p.Arg408Trp mutation is proposed to have a dominating negative effect [20]. One cPKU patient with c. [1199 + 1G > C]; [1066-11G > A] genotype showed a 37.5 % Phe concentration decrease (H0 1152 μmol/L; tested between 10 and 18 yo) but 1 mPKU ((H0 822 μmol/L; tested between 3 and 10 yo) and 1 cPKU (H0 3258 μmol/L; tested as neonate) with the same genotype were non-responsive. In addition, one homozygote c. [1199 + 1G > C]; [1199 + 1G > C] cPKU were non-responsive (H0 1842 μmol/L; tested at adulthood). Finally, c. [493G > A]; [493G > A] genotype was observed in two cPKU siblings. The one tested between 3 and 10 years of age showed no decrease in Phe concentration (H0 384 μmol/L) while the one tested in the neonatal period showed a 36 % decrease (H0 1680 μmol/L) but is not currently under BH4 treatment.

Conversely, 6 nonsense mutations – coding for p.Glu57*, p.Glu66*, p.Tyr168*; p.Tyr216*, p.Asp222*, p.Glu280*, and the deletion c.1055delG (p.Gly352Vfs*48), observed in 7 homozygous patients with no response to BH4 challenging, were considered as BH4-unresponsive.

### Genotype-phenotype associations

In Table 1, the BH4-loading tests for the 261 tabulated genotypes are listed together with the relative frequency and associated PKU phenotypes. Most of the genotypes were associated with a unique class of PKU severity. However, c. [194 T > C]; [1208C > T] (p.[Ile65Thr]; [Ala403Val], *n* = 3) and c. [1208C > T]; [1222C > T] (p.[Ala403Val]; [Arg408Trp], *n* = 2) genotypes were associated with both mHP and mPKU and 12 other genotypes (*n* = 36) were observed in both mPKU and cPKU. In addition, c. [1223G > A]; [1241A > G] (p.[Arg408Gln]; [Tyr414Cys], *n* = 2) was inconsistently associated with mHP and cPKU.

**Table 1:** Genotypes observed in French patients with phenylketonuria. Association with clinical phenotypes and response to BH4 loading-test

Genotypes (?: neither mutation found with sequencing nor rearrangement found in MLPA; #: newly described mutation)		Number (freq %)	Number of BH4-loading tests (responders/non responders)			Total BH4 loading test responders	Total BH4 loading test non-responders	Number of responsive and/or non-responsive patients previously recorded in the BioPKU database for each responsive genotype of the present study	Predicted BH4-sensitivity (PRA) according to the literature ([10] except ^a^ [9]; ^b^ [30]; c.1066-11G > A [2])			Responsiveness as proposed in this manuscript if "Predicted BH4-sensitivity according to the literature" = unk	
			mHP	mPKU	cPKU				mut1	mut2	mut3	mut1	mut2
c. [1A > G]; [782G > A]	p. [Met1Val]; [Arg261Gln]	1 (0.38)	-	-	1 (0/1)	0	1 (cPKU)		unk	R^a^ (38.5)	-		
c. [60G > C]; [473G > A]	p. [Gln20His]; [Arg158Gln]	1 (0.38)	-	-	1 (0/1)	0	1 (cPKU)		unk	R (10)	-		
c. [60 + 5G > A]^#^; [1042C > G]	p. [?]; [Leu348Val]	1 (0.38)	-	-	1 (0/1)	0	1 (cPKU)		unk	R (41)	-		
c. [115_117delTTC]; [1042C > G]	p. [Phe39del]; [Leu348Val]	1 (0.38)	-	-	1 (0/1)	0	1 (cPKU)		R (20)	R (41)	-		
c. [115_117delTTC]; [1315 + 1G > A]	p. [Phe39del]; [?]	2 (0.77)	-	-	2 (0/2)	0	2 (2 cPKU)		R (20)	NR (0)	-		
c. [117C > G]; [785 T > G]^#^	p. [Phe39Leu]; [Val262Gly]	1 (0.38)	-	-	1 (0/1)	0	1 (cPKU)		R (49)	unk	-		
c. [117C > G]; [814G > T]	p. [Phe39Leu]; [Gly272*]	1 (0.38)	-	-	1 (0/1)	0	1 (cPKU)		R (49)	NR (1)	-		
c. [117C > G]; [1169A > G]	p. [Phe39Leu]; [Glu390Gly]	1 (0.38)	-	-	1 (1/0)	1 (cPKU)	0	no data	R (49)	R (72.5)	-		
c. [143 T > C]; [143 T > C]	p. [Leu48Ser]; [Leu48Ser]	3 (1.15)	-	2 (2/0)	1 (1/0)	3 (2 mPKU, 1cPKU)	0	37 R; 5 NR	R (39)	R (39)	-		
c. [143 T > C]; [165delT]	p. [Leu48Ser]; [Phe55Leufs*6]	2 (0.77)	-	-	2 (2/0)	2 (cPKU)	0	2 PKU R	R (39)	unk	-		
c. [143 T > C]; [526C > T]	p. [Leu48Ser]; [Arg176*]	1 (0.38)	-	-	1 (1/0)	1 (cPKU)	0	no data	R (39)	NR (1)	-		
c. [143 T > C]; [547_548delinsTT]^#^	p. [Leu48Ser]; [Glu183Leu]	1 (0.38)	-	-	1 (0/1)	0	1 (cPKU)		R (39)	unk	-		
c. [143 T > C]; [782G > A]	p. [Leu48Ser]; [Arg261Gln]	2 (0.77)	-	-	2 (2/0)	2 (cPKU)	0	22 R; 5 NR	R (39)	R^a^ (38,5)	-		
c. [143 T > C]; [1045 T > C]	p. [Leu48Ser]; [Ser349Pro]	1 (0.38)	-	-	1 (0/1)	0	1 (cPKU)		R (39)	NR (1)	-		
c. [143 T > C]; [1055delG]	p. [Leu48Ser]; [Gly352Valfs*48]	1 (0.38)	-	-	1 (0/1)	0	1 (cPKU)		R (39)	unk	-		NR
c. [143 T > C]; [1066-11G > A]	p. [Leu48Ser]; [?]	3 (1.15)	-	1 (0/1)	2 (0/2)	0	3 (1 mPKU, 2 cPKU)		R (39)	NR (0)	-		
c. [143 T > C]; [1162G > A]	p. [Leu48Ser]; [Val388Met]	1 (0.38)	-	1 (1/0)	-	1 (mPKU)	0	2 mPKU R	R (39)	R (27,5)	-		
c. [143 T > C]; [442-?_509 + ?del]	p. [Leu48Ser]; [Ex5del]	1 (0.38)	-	-	1 (0/1)	0	1 (cPKU)		R (39)	unk	-		
c. [155delT]^#^; [781C > T]	p. [Leu52Cysfs*9]; [Arg261*]	1 (0.38)	-	-	1 (0/1)	0	1 (cPKU)		unk	NR (1)	-		
c. [158G > A]; [?]	p. [Arg53His]; [?]	1 (0.38)	-	1 (1/0)	-	1 (mPKU)	0		unk (79)	-	-		
c. [164 T > C]^#^; [1066-11G > A]	p. [Phe55Ser]; [?]	1 (0.38)	-	-	1 (0/1)	0	1 (cPKU)		unk	NR (0)	-		
c. [165delT]; [194 T > C]	p. [Phe55Leufs*6]; [Ile65Thr]	1 (0.38)	-	-	1 (0/1)	0	1 (cPKU)		unk	R (25,3)	-		
c. [165delT]; [441 + 5G > T]	p. [Phe55Leufs*6]; [?]	2 (0.77)	-	-	2 (0/2)	0	2 (2 cPKU)		unk	NR^b^	-		
c. [165delT]; [727C > T]	p. [Phe55Leufs*6]; [Arg243*]	1 (0.38)	-	-	1 (0/1)	0	1 (cPKU)		unk	NR (1)	-		
c. [165delT]; [1066-11G > A]	p. [Phe55Leufs*6]; [?]	1 (0.38)	-	-	1 (0/1)	0	1 (cPKU)		unk	NR (0)	-		
c. [165delT]; [1315 + 1G > A]	p. [Phe55Leufs*6]; [?]	1 (0.38)	-	-	1 (0/1)	0	1 (cPKU)		unk	NR (0)	-		
c. [168 + 5G > C]; [168 + 5G > C]	p. [?]; [?]	1 (0.38)	-	-	1 (0/1)	0	1 (cPKU)		unk	unk	-		
c. [168 + 5G > C]; [782G > A]	p. [?]; [Arg261Gln]	2 (0.77)	-	-	2 (1/1)	1 (cPKU) minus 61 %	1 (cPKU)	3 PKU R; 1 PKU NR	unk	R^a^ (38.5)	-		
c. [168 + 5G > C]; [1-?_60 + ?del]^#^	p. [?]; [Ex1del]	1 (0.38)	-	-	1 (0/1)	0	1 (cPKU)		unk	unk	-		
c. [169G > T]^#^; [194 T > C]	p. [Glu57*]; [Ile65Thr]	1 (0.38)	-	-	1 (0/1)	0	1 (cPKU)		unk	R (25,3)	-	NR	
c. [194 T > C]; [204A > T]	p. [Ile65Thr]; [Arg68Ser]	1 (0.38)	-	1 (1/0)	-	1 (mPKU)	0	2 PKU R; 1 PKU NR	R (25.3)	R (87)	-		
c. [194 T > C]; [331C > T]	p. [Ile65Thr]; [Arg111*]	1 (0.38)	-	-	1 (0/1)	0	1 (cPKU)		R (25.3)	NR (1)	-		
c. [194 T > C]; [386A > G]	p. [Ile65Thr]; [Asp129Gly]	1 (0.38)	-	-	1 (0/1)	0	1 (cPKU)		R (25.3)	R	-		
c. [194 T > C]; [473G > A]	p. [Ile65Thr]; [Arg158Gln]	1 (0.38)	-	-	1 (0/1)	0	1 (cPKU)		R (25.3)	R (10)	-		
c. [194 T > C]; [526C > T]	p. [Ile65Thr]; [Arg176*]	1 (0.38)	-	-	1 (0/1)	0	1 (cPKU)		R (25.3)	NR (1)	-		
c. [194 T > C]; [782G > A]	p. [Ile65Thr]; [Arg261Gln]	3 (1.15)	-	-	3 (0/3)	0	3 (3 cPKU)		R (25.3)	R^a^ (38,5)	-		
c. [194 T > C]; [782G > C]	p. [Ile65Thr]; [Arg261Pro]	1 (0.38)	-	-	1 (0/1)	0	1 (cPKU)		R (25.3)	unk	-		
c. [194 T > C]; [829 T > G]	p. [Ile65Thr]; [Tyr277Asp]	1 (0.38)	-	1 (0/1)	-	0	1 (mPKU)		R (25.3)	unk (0–12)	-		
c. [194 T > C]; [838G > A]	p. [Ile65Thr]; [Glu280Lys]	1 (0.38)	-	-	1 (0/1)	0	1 (cPKU)		R (25.3)	NR (1.95)	-		
c. [194 T > C]; [842C > T]	p. [Ile65Thr]; [Pro281Leu]	2 (0.77)	-	-	2 (0/2)	0	2 (2 cPKU)		R (25.3)	NR (1)	-		
c. [194 T > C]; [842 + 1G > A]	p. [Ile65Thr]; [?]	1 (0.38)	-	-	1 (0/1)	0	1 (cPKU)		R (25.3)	unk	-		
c. [194 T > C]; [844G > A]	p. [Ile65Thr]; [Asp282Asn]	1 (0.38)	-	-	1 (0/1)	0	1 (cPKU)		R (25.3)	unk (2)	-		
c. [194 T > C]; [898G > T]	p. [Ile65Thr]; [Ala300Ser]	1 (0.38)	1 (1/0)	-	-	1 (mHP)	0	2 mHP R	R (25.3)	R (31)	-		
c. [194 T > C]; [912 + 1G > A]	p. [Ile65Thr]; [?]	1 (0.38)	-	-	1 (0/1)	0	1 (cPKU)		R (25.3)	unk	-		
c. [194 T > C]; [916A > G]	p. [Ile65Thr]; [I306Val]	1 (0.38)	-	1 (1/0)	-	1 (mPKU)	0	2 R	R (25.3)	unk (12–39)	-		
c. [194 T > C]; [932 T > C]	p. [Ile65Thr]; [Leu311Pro]	1 (0.38)	-	-	1 (0/1)	0	1 (cPKU)		R (25.3)	NR (1)	-		
c. [194 T > C]; [1042C > G]	p. [Ile65Thr]; [Leu348Val]	1 (0.38)	-	1 (1/0)	-	1 (mPKU)	0	5 PKU R; 1PKU NR	R (25.3)	R (41)	-		
c. [194 T > C]; [1045 T > C]	p. [Ile65Thr]; [Ser349Pro]	1 (0.38)	-	-	1 (0/1)	0	1 (cPKU)		R (25.3)	NR (1)	-		
c. [194 T > C]; [1066-11G > A]	p. [Ile65Thr]; [?]	4 (1.53)	-	-	4 (0/4)	0	4 (4 cPKU)		R (25.3)	NR (0)	-		
c. [194 T > C]; [1169A > G]	p. [Ile65Thr]; [Glu390Gly]	2 (0.77)	-	1 (1/0)	1 (1/0)	2 (mPKU, cPKU)	0	3 PKU R	R (25.3)	R (72.5)	-		
c. [194 T > C]; [1169A > G]; [1171A > G]^#^	p. [Ile65Thr]; ([Glu390Gly)]; [(Ser391Gly)]	1 (0.38)	-	-	1 (1/0)	1 (cPKU)	0		R (25.3)	R (72.5)	unk		
c. [194 T > C]; [1208C > T]	p. [Ile65Thr]; [Ala403Val]	4 (1.53)	2 (2/0)	1 (0/1)	-	2 (2 mHP)	1 (PKUa) minus 15 %	1 mHP R	R (25.3)	R (32)	-		
c. [194 T > C]; [1222C > T]	p. [Ile65Thr]; [Arg408Trp]	2 (0.77)	-	-	2 (1/1)	1 (cPKU) minus 32 %	1 (cPKU) minus 14 %	9 R; 8 NR	R (25.3)	NR (1)	-		
c. [196G > T]^#^; [1315 + 1G > A]	p. [Glu66*]; [?]	1 (0.38)	-	-	1 (0/1)	0	1 (cPKU)		unk	NR (0)	-	NR	
c. [204A > T]; [204A > T]	p. [Arg68Ser]; [Arg68Ser]	1 (0.38)	-	1 (1/0)	-	1 (mPKU)	0	1 mPKU R	R (87)	R (87)	-		
c. [204A > T]; [398_401delATCA]	p. [Arg68Ser]; [Asn133Argfs*61]	1 (0.38)	-	-	1 (0/1)	0	1 (cPKU)		R (87)	unk	-		
c. [204A > T]; [526C > T]; [707-7A > T]	p. [Arg68Ser]; [(Arg176*)]; [(?)]	1 (0.38)	-	1 (1/0)	-	1 (mPKU)	0	no data	R (87)	NR (1)	unk		
c. [204A > T]; [611A > G]	p. [Arg68Ser]; [Tyr204Cys]	1 (0.38)	-	1 (0/1)	-	0	1 (mPKU)		R (87)	unk	-		
c. [204A > T]; [814G > T]	p. [Arg68Ser]; [Gly272*]	1 (0.38)	-	1 (1/0)	-	1 (mPKU)	0	1cPKU R	R (87)	NR (1)	-		
c. [204A > T]; [1045 T > C]	p. [Arg68Ser]; [Ser349Pro]	1 (0.38)	1 (1/0)	-	-	1 (mHP)	0	no data	R (87)	NR (1)	-		
c. [204A > T]; [1055delG]	p. [Arg68Ser]; [Gly352Valfs*48]	1 (0.38)	-	1 (0/1)	-	0	1 (mPKU)		R (87)	unk	-		NR
c. [204A > T]; [1162G > A]	p. [Arg68Ser]; [Val388Met]	1 (0.38)	1 (1/0)	-	-	1 (mHP)	0	no data	R (87)	R (27.5)	-		
c. [204A > T]; [1222C > T]	p. [Arg68Ser]; [Arg408Trp]	2 (0.77)	-	1 (0/1)	1 (0/1)	0	2 (mPKU, cPKU)		R (87)	NR (1)	-		
c. [204A > T]; [1315 + 1G > A]	p. [Arg68Ser]; [?]	1 (0.38)	-	1 (0/1)	-	0	1 (mPKU)		R (87)	NR (0)	-		
c. [261C > A]; [1065 + 1G > A]	p. [Ser87Arg]; [?]	1 (0.38)	1 (0/1)	-	-	0	1 (mHP, H0 2.4 mg %, minus 8 %)		R (82)	unk	-		
c. [266_267insC]; [842C > T]	p. [Ala90Cysfs*12]; [Pro281Leu]	1 (0.38)	-	-	1 (1/0)	1 (cPKU) minus 36 %	0	no data	unk	NR (1)	-	unclear	
c. [284_286delTCA]; [441 + 5G > T]	p. [Ile95del]; [?]	1 (0.38)	-	-	1 (0/1)	0	1 (cPKU)		unk	NR^b^	-		
c. [284_286delTCA]; [1243G > A]	p. [Ile95del]; [Asp415Asn]	1 (0.38)	1 (1/0)	-	-	1 (mHP)	0	no data	unk	R (93)	-		
c. [311C > A]; [442-5C > G]	p. [Ala104Asp]; [?]	1 (0.38)	-	1 (0/1)	-	0	1 (mPKU)		R (26)	R	-		
c. [311C > A]; [1222C > T]	p. [Ala104Asp]; [Arg408Trp]	1 (0.38)	-	-	1 (0/1)	0	1 (cPKU)		R (26)	NR (1)	-		
c. [331C > T]; [782G > A]	p. [Arg111*]; [Arg261Gln]	1 (0.38)	-	-	1 (0/1)	0	1 (cPKU)		NR (1)	R^a^ (38.5)	-		
c. [331C > T]; [1222C > T]	p. [Arg111*]; [Arg408Trp]	1 (0.38)	-	-	1 (0/1)	0	1 (cPKU)		NR (1)	NR (1)	-		
c. [434A > T]; [1222C > T]	p. [Asp145Val]; [Arg408Trp]	1 (0.38)	1 (1/0)	-	-	1 (mHP)	0	no data	unk	NR (1)	-	R	
c. [441 + 5G > T]; [441 + 5G > T]	p. [?]; [?]	1 (0.38)	-	-	1 (0/1)	0	1 (cPKU)		NR^b^	NR^b^	-		
c. [441 + 5G > T]; [473G > A]	p. [?]; [Arg158Gln]	2 (0.77)	-	-	2 (0/2)	0	2 (2 cPKU)		NR^b^	R (10)	-		
c. [441 + 5G > T]; [631C > A]	p. [?]; [Pro211Thr]	1 (0.38)	1 (1/0)	-	-	1 (mHP)	0	no data	NR^b^	R (72)	-		
c. [441 + 5G > T]; [782G > A]	p. [?]; [Arg261Gln]	2 (0.77)	-	-	2 (0/2)	0	2 (2 cPKU)		NR^b^	R^a^ (38.5)	-		
c. [441 + 5G > T]; [838G > A]	p. [?]; [Glu280Lys]	1 (0.38)	-	-	1 (0/1)	0	1 (cPKU)		NR_b_	NR (1.95)	-		
c. [441 + 5G > T]; [1045 T > G]	p. [?]; [Ser349Ala]	1 (0.38)	-	-	1 (1/0)	1 (cPKU) minus 41 %	0	no data	NR_b_	unk	-		R
c. [442-2A > C]^#^; [442-2A > C]^#^	p. [?]; [?]	1 (0.38)	-	-	1 (0/1)	0	1 (cPKU)		unk	unk	-		
c. [464G > C]; [1223G > A]	p. [Arg155Pro]; [Arg408Gln]	1 (0.38)	-	-	1 (0/1)	0	1 (cPKU)		unk	R (49.7)	-		
c. [464G > C]; [510-?-706 + ?del]	p. [Arg155Pro]; [Ex6del]	1 (0.38)	-	-	1 (0/1)	0	1 (cPKU)		unk	unk	-		
c. [472C > T]; [472C > T]	p. [Arg158Trp]; [Arg158Trp]	1 (0.38)	-	1 (0/1)	-	0	1 (mPKU)		unk	unk	-		
c. [472C > T]; [754C > T]	p. [Arg158Trp]; [Arg252Trp]	1 (0.38)	-	-	1 (0/1)	0	1 (cPKU)		unk	NR (1)	-		
c. [473G > A]; [473G > A]	p. [Arg158Gln]; [Arg158Gln]	1 (0.38)	-	-	1 (0/1)	0	1 (cPKU)		R (10)	R (10)	-		
c. [473G > A]; [526C > T]	p. [Arg158Gln]; [Arg176*]	1 (0.38)	-	-	1 (0/1)	0	1 (cPKU)		R (10)	NR (1)	-		
c. [473G > A]; [814G > T]	p. [Arg158Gln]; [Gly272*]	1 (0.38)	-	-	1 (0/1)	0	1 (cPKU)		R (10)	NR (1)	-		
c. [473G > A]; [1066-11G > A]	p. [Arg158Gln]; [?]	3 (1.15)	-	-	3 (0/3)	0	3 (3 cPKU)		R (10)	NR (0)	-		
c. [473G > A]; [1169A > G]	p. [Arg158Gln]; [Glu390Gly]	1 (0.38)	-	1 (1/0)	-	1 (mPKU)	0	5 R	R (10)	R (72.5)	-		
c. [473G > A]; [1222C > T]	p. [Arg158Gln]; [Arg408Trp]	1 (0.38)	-	-	1 (0/1)	0	1 (cPKU)		R (10)	NR (1)	-		
c. [473G > A]; [169-?_352 + ?del]	p. [Arg158Gln]; [Ex3del]	1 (0.38)	-	-	1 (0/1)	0	1 (cPKU)		R (10)	unk	-		
c. [473G > T]; [782G > C]	p. [Arg158Trp]; [Arg261Pro]	1 (0.38)	-	-	1 (0/1)	0	1 (cPKU)		unk	unk	-		
c. [491 T > C]; [1241A > G]	p. [Ile164Thr]; [Tyr414Cys]	1 (0.38)	-	1 (1/0)	-	1 (mPKU)	0	no data	unk	R^a^ (36)	-		
c. [493G > A]; [493G > A]	p. [Ala165Thr]; [Ala165Thr]	2 (0.77)	-	-	2 (1/1)	1 (cPKU) minus 36 %	1 (cPKU) minus 0 %	no data	unk	unk	-	unclear	unclear
c. [493G > C]; [1169A > G]	p. [Ala165Pro]; [Glu390Gly]	1 (0.38)	-	1 (1/0)	-	1 (mPKU)	0	no data	unk	R (72.5)	-		
c. [500A > T]; [1066-11G > A]	p. [Asn167Ile]; [?]	1 (0.38)	-	1 (1/0)	-	1 (mPKU)	0	no data	unk	NR (0)	-	R	
c. [504C > A]^#^; [898G > T]	p. [Tyr168*]; [Ala300Ser]	1 (0.38)	1 (1/0)	-	-	1 (mHP)	0	no data	unk	R (31)	-	NR	
c. [506G > A]; [1066-3C > T]	p. [Arg169His]; [?]	1 (0.38)	1 (1/0)	-	-	1 (mHP)	0	no data	unk	R	-		
c. [509 + 1G > A]; [561G > A]	p. [?]; [Trp187*]	1 (0.38)	-	-	1 (0/1)	0	1 (cPKU)		unk	NR (0)	-		
c. [529G > C]; [561G > A]	p. [Val177Leu]; [Trp187*]	1 (0.38)	-	1 (1/0)	-	1 (mPKU)	0	no data	unk	NR (0)	-	R	
c. [569 T > C]; [796A > C]^#^	p. [Val190Ala]; [Thr266P]	1 (0.38)	-	-	1 (0/1)	0	1 (cPKU)		R (110)	unk	-		
c. [580_581delCT]; [1042C > G]	p. [Leu194Glufs*5]; [Leu348Val]	1 (0.38)	-	-	1 (0/1)	0	1 (cPKU)		unk	R (41)	-		
c. [581 T > C]; [842C > T]	p. [Leu194Pro]; [Pro281Leu]	1 (0.38)	-	1 (0/1)	-	0	1 (mPKU)		unk	NR (1)	-		
c. [593_614del22]; [969 + 1G > A]	p. [Tyr198Cysfs*136]; [?]	1 (0.38)	-	-	1 (0/1)	0	1 (cPKU)		unk	unk	-		
c. [631C > A]; [782G > A]	p. [Pro211Thr]; [Arg261Gln]	2 (0.77)	-	1 (1/0)	1 (1/0)	2 (mPKU, cPKU)	0	4 mHP R	R (72)	R^a^ (38,5)	-		
c. [631C > A]; [1066-11G > A]	p. [Pro211Thr]; [?]	1 (0.38)	-	1 (1/0)	-	1 (mPKU)	0	10 R	R (72)	NR (0)	-		
c. [632delC]; [1066-11G > A]	p. [Pro211Hisfs*130]; [?]	1 (0.38)	-	-	1 (0/1)	0	1 (cPKU)		unk	NR (0)	-		
c. [635 T > C]; [1066-11G > A]	p. [Leu212Pro]; [?]	1 (0.38)	-	-	1 (0/1)	0	1 (cPKU)		unk	NR (0)	-		
c. [638 T > C]; [638 T > C]	p. [Leu213Pro]; [Leu213Pro]	1 (0.38)	-	-	1 (0/1)	0	1 (cPKU)		unk	unk	-		
c. [638 T > C]; [727C > T]	p. [Leu213Pro]; [Arg243*]	1 (0.38)	-	-	1 (0/1)	0	1 (cPKU)		unk	NR (1)	-		
c. [648C > G]; [838G > A]	p. [Tyr216*]; [Glu280Lys]	1 (0.38)	-	-	1 (0/1)	0	1 (cPKU)		unk	NR (1.95)	-	NR	
c. [653G > T]; [809G > A]	p. [Gly218Val]; [Arg270Lys]	1 (0.38)	-	-	1 (0/1)	0	1 (cPKU)		unk	NR (2.1)	-		
c. [653G > T]; [1066-11G > A]	p. [Gly218Val]; [?]	1 (0.38)	-	-	1 (0/1)	0	1 (cPKU)		unk	NR (0)	-		
c. [663_664delAG]; [912 + 1G > A]	p. [Asp222*]; [?]	1 (0.38)	-	-	1 (0/1)	0	1 (cPKU)		NR (1)	unk	-		
c. [671 T > C]; [671 T > C]	p. [Ile224Thr]; [Ile224Thr]	2 (0.77)	-	-	2 (0/2)	0	2 (2 cPKU)		unk	unk	-		
c. [682G > A]^#^; [1066-11G > A]	p. [Glu228Lys]; [?]	1 (0.38)	-	-	1 (0/1)	0	1 (cPKU)		unk	NR (0)	-		
c. [721C > T]; [931_932delCT]^#^	p. [Arg241Cys]; [Leu311Glyfs*4]	1 (0.38)	-	1 (0/1)	-	0	1 (mPKU)		R (25)	unk	-		
c. [722G > A]; [722G > A]	p. [Arg241His]; [Arg241His]	1 (0.38)	1 (1/0)	-	-	1 (mHP)	0	no data	R (23)	R (23)	-		
c. [722G > A]; [782G > A]	p. [Arg241His]; [Arg261Gln]	1 (0.38)	1 (1/0)	-	-	1 (mHP)	0	no data	R (23)	R^a^ (38,5)	-		
c. [722G > A]; [838_842 + 3del8]	p. [Arg241His]; [His280*]	1 (0.38)	1 (1/0)	-	-	1 (mHP)	0	no data	R (23)	unk	-		NR
c. [722G > A]; [842 + 1G > A]	p. [Arg241His]; [?]	1 (0.38)	-	1 (1/0)	-	1 (mPKU)	0	no data	R (23)	unk	-		
c. [722G > A]; [1066-11G > A]	p. [Arg241His]; [?]	1 (0.38)	-	1 (1/0)	-	1 (mPKU)	0	no data	R (23)	NR (0)	-		
c. [727C > T]; [727C > T]	p. [Arg243*]; [Arg243*]	1 (0.38)	-	-	1 (0/1)	0	1 (cPKU)		NR (1)	NR (1)	-		
c. [727C > T]; [842C > T]	p. [Arg243*]; [Pro281Leu]	1 (0.38)	-	-	1 (0/1)	0	1 (cPKU)		NR (1)	NR (1)	-		
c. [727C > T]; [912 + 1G > A]	p. [Arg243*]; [?]	1 (0.38)	-	-	1 (0/1)	0	1 (cPKU)		NR (1)	unk	-		
c. [727C > T]; [1042C > G]	p. [Arg243*]; [Leu348Val]	1 (0.38)	-	-	1 (0/1)	0	1 (cPKU)		NR (1)	R (41)	-		
c. [727C > T]; [1066-11G > A]	p. [Arg243*]; [?]	1 (0.38)	-	-	1 (0/1)	0	1 (cPKU)		NR (1)	NR (0)	-		
c. [727C > T]; [?]	p. [Arg243*]; [?]	1 (0.38)	-	-	1 (0/1)	0	1 (cPKU)		NR (1)	-	-		
c. [728G > A]; [913-7A > G]	p. [Arg243Gln]; [?]	1 (0.38)	-	-	-	0	1 (unk)		R (23)	unk	-		
c. [728G > A]; [912 + 1G > A]	p. [Arg243Gln]; [?]	2 (0.77)	-	-	2 (0/2)	0	2 (2 cPKU)		R (23)	unk	-		
c. [728G > A]; [1055delG]	p. [Arg243Gln]; [Gly352Valfs*48]	2 (0.77)	-	-	2 (0/2)	0	2 (2 cPKU)		R (23)	unk	-		NR
c. [728G > A]; [1208C > T]	p. [Arg243Gln]; [Ala403Val]	1 (0.38)	1 (1/0)	-	-	1 (mHP)	0	6 mHP R	R (23)	R (32)	-		
c. [734 T > C]; [1315 + 1G > A]	p. [Val245Ala]; [?]	1 (0.38)	1 (0/1)	-	-	0	1 (mHP, H0 2.4 mg %, minus 4 %)		R (50)	NR (0)	-		
c. [754C > T]; [838G > A]	p. [Arg252Trp]; [Glu280Lys]	1 (0.38)	-	-	1 (0/1)	0	1 (cPKU)		NR (1)	NR (1.95)	-		
c. [754C > T]; [838_842 + 3del8]	p. [Arg252Trp]; [His280*]	1 (0.38)	-	-	1 (0/1)	0	1 (cPKU)		NR (1)	unk	-		NR
c. [754C > T]; [1042C > G]	p. [Arg252Trp]; [Leu348Val]	1 (0.38)	-	-	1 (0/1)	0	1 (cPKU)		NR (1)	R (41)	-		
c. [754C > T]; [1045 T > G]	p. [Arg252Trp]; [Ser349Ala]	1 (0.38)	-	1 (1/0)	-	1 (mPKU) minus 51 %	0	no data	NR (1)	unk	-		R
c. [754C > T]; [1055delG]	p. [Arg252Trp]; [Gly352Valfs*48]	1 (0.38)	-	-	1 (0/1)	0	1 (cPKU)		NR (1)	unk	-		NR
c. [754C > T]; [1066-11G > A]	p. [Arg252Trp]; [?]	1 (0.38)	-	-	1 (0/1)	0	1 (cPKU)		NR (1)	NR (0)	-		
c. [754C > T]; [1208C > T]	p. [Arg252Trp]; [Ala403Val]	1 (0.38)	-	1 (1/0)	-	1 (mPKU)	0	3 R	NR (1)	R (32)	-		
c. [754C > T]; [1241A > G]	p. [Arg252Trp]; [Tyr414Cys]	1 (0.38)	-	-	1 (1/0)	1 (cPKU)	0	4 R	NR (1)	R^a^ (36)	-		
c. [755G > A]; [782G > A]	p. [Arg252Gln]; [Arg261Gln]	1 (0.38)	-	-	1 (1/0)	1 (cPKU)	0	no data	unk (3–24)	R^a^ (38,5)	-		
c. [755G > A]; [1066-11G > A]	p. [Arg252Gln]; [?]	1 (0.38)	-	-	1 (0/1)	0	1 (cPKU)		unk (3–24)	NR (0)	-		
c. [770G > A]; [932 T > C]	p. [Gly257Asp]; [Leu311Pro]	1 (0.38)	-	-	1 (0/1)	0	1 (cPKU)		unk	NR (1)	-		
c. [776C > T]; [1066-11G > A]	p. [Ala259Val]; [?]	2 (0.77)	-	-	2 (0/2)	0	2 (2 cPKU)		unk (2)	NR (0)	-		
c. [776C > T]; [1222C > T]	p. [Ala259Val]; [Arg408Trp]	2 (0.77)	-	-	2 (0/2)	0	2 (2 cPKU)		unk (2)	NR (1)	-		
c. [776C > T]; [970-?_1359 + ?]^#^	p. [Ala259Val]; [Ex10_13del]	1 (0.38)	-	-	1 (0/1)	0	1 (cPKU)		unk (2)	unk	-		
c. [781C > G]^#^; [913-7A > G]	p. [Arg261Gly]; [?]	1 (0.38)	-	-	1 (0/1)	0	1 (cPKU)		unk	unk	-		
c. [781C > T]; [781C > T]	p. [Arg261*]; [Arg261*]	1 (0.38)	-	-	1 (0/1)	0	1 (cPKU)		NR (1)	NR (1)	-		
c. [781C > T]; [782G > A]	p. [Arg261*]; [Arg261Gln]	1 (0.38)	-	-	1 (0/1)	0	1 (cPKU)		NR (1)	R^a^ (38.5)	-		
c. [781C > T]; [1222C > T]	p. [Arg261*]; [Arg408Trp]	1 (0.38)	-	-	1 (0/1)	0	1 (cPKU)		NR (1)	NR (1)	-		
c. [782G > A]; [782G > A]	p. [Arg261Gln]; [Arg261Gln]	8 (3.065)	-	-	8 (6/2)	6 (cPKU)	2 (cPKU)	59 R; 11 NR	R^a^ (38,5)	R^a^ (38,5)	-		
c. [782G > A]; [782G > C]	p. [Arg261Gln]; [Arg261Pro]	1 (0.38)	-	-	1 (0/1)	0	1 (cPKU)		R^a^ (38,5)	unk	-		
c. [782G > A]; [814G > T]	p. [Arg261Gln]; [Gly272*]	2 (0.77)	-	-	2 (0/2)	0	2 (2 cPKU)		R^a^ (38,5)	NR (1)	-		
c. [782G > A]; [838G > A]	p. [Arg261Gln]; [Glu280Lys]	1 (0.38)	-	-	1 (0/1)	0	1 (cPKU)		R^a^ (38,5)	NR (1.95)	-		
c. [782G > A]; [842 + 1G > A]	p. [Arg261Gln]; [?]	2 (0.77)	-	-	2 (0/2)	0	2 (2 cPKU)		R^a^ (38,5)	unk	-		
c. [782G > A]; [912 + 1G > A]	p. [Arg261Gln]; [?]	1 (0.38)	-	-	1 (0/1)	0	1 (cPKU)		R^a^ (38,5)	unk	-		
c. [782G > A]; [1045 T > C]	p. [Arg261Gln]; [Ser349Pro]	2 (0.77)	-	-	2 (1/1)	1 (cPKU) minus 41 %	1 (cPKU) minus 20 %	3 R	R^a^ (38,5)	NR (1)	-		
c. [782G > A]; [1055delG]	p. [Arg261Gln]; [Gly352Valfs*48]	1 (0.38)	-	-	1 (0/1)	0	1 (cPKU)		R^a^ (38,5)	unk	-		NR
c. [782G > A]; [1066-11G > A]	p. [Arg261Gln]; [?]	4 (1.53)	-	-	4 (0/4)	0	4 (4 cPKU)		R^a^ (38,5)	NR (0)	-		
c. [782G > A]; [1068C > G]	p. [Arg261Gln]; [Tyr356*]	1 (0.38)	-	-	1 (0/1)	0	1 (cPKU)		R^a^ (38,5)	NR (1)	-		
c. [782G > A]; [1162G > A]	p. [Arg261Gln]; [Val388Met]	1 (0.38)	-	-	1 (0/1)	0	1 (cPKU)		R^a^ (38,5)	R (72.5)	-		
c. [782G > A]; [1208C > T]	p. [Arg261Gln]; [Ala403Val]	1 (0.38)	1 (1/0)	-	-	1 (mHP)	0	6 mHP R	R^a^ (38,5)	R (32)	-		
c. [782G > A]; [1222C > T]	p. [Arg261Gln]; [Arg408Trp]	4 (1.53)	-	1 (0/1)	3 (0/3)	0	4 (1 mPKU, 3 cPKU)		R^a^ (38,5)	NR (1)	-		
c. [782G > A]; [1241A > G]	p. [Arg261Gln]; [Tyr414Cys]	1 (0.38)	-	1 (1/0)	-	1 (mPKU)	0	12 R	R^a^ (38,5)	R^a^ (38,5)	-		
c. [782G > A]; [1315 + 1G > A]	p. [Arg261Gln]; [?]	5 (1.915)	-	-	5 (0/5)	0	5 (5 cPKU)		R^a^ (38,5)	NR (0)	-		
c. [782G > A]; [510-?-706 + ?del]	p. [Arg261Gln]; [Ex6del]	1 (0.38)	-	-	1 (0/1)	0	1 (cPKU)		R^a^ (38,5)	unk	-		
c. [782G > C]; [1241A > G]	p. [Arg261Pro]; [Tyr414Cys]	1 (0.38)	-	-	1 (1/0)	1 (cPKU)	0	2 PKU R; 1 PKU NR	unk	R^a^ (36)	-		
c. [782G > C]; [1315 + 1G > A]	p. [Arg261Pro]; [?]	1 (0.38)	-	-	1 (0/1)	0	1 (cPKU)		unk	NR (0)	-		
c. [812A > G]; [1222C > T]	p. [His271Arg]; [Arg408Trp]	1 (0.38)	-	-	1 (0/1)	0	1 (cPKU)		unk	NR (1)	-		
c. [814G > T]; [1042C > G]	p. [Gly272*]; [Leu348Val]	1 (0.38)	-	-	1 (0/1)	0	1 (cPKU)		NR (1)	R (41)	-		
c. [814G > T]; [1045 T > C]	p. [Gly272*]; [Ser349Pro]	1 (0.38)	-	-	1 (0/1)	0	1 (cPKU)		NR (1)	NR (1)	-		
c. [814G > T]; [1066-11G > A]	p. [Gly272*]; [?]	1 (0.38)	-	-	1 (0/1)	0	1 (cPKU)		NR (1)	NR (0)	-		
c. [814G > T]; [1162G > A]	p. [Gly272*]; [Val388Met]	1 (0.38)	-	-	1 (1/0)	1 (cPKU)	0	no data	NR (1)	R (27.5)	-		
c. [814G > T]; [1169A > G]	p. [Gly272*]; [Glu390Gly]	2 (0.77)	-	2 (2/0)	-	2 (mPKU)	0	2 R	NR (1)	R (72.5)	-		
c. [814G > T]; [1208C > T]	p. [Gly272*]; [Ala403Val]	1 (0.38)	1 (1/0)	-	-	1 (mHP)	0	no data	NR (1)	R (32)	-		
c. [814G > T]; [1222C > T]	p. [Gly272*]; [Arg408Trp]	1 (0.38)	-	-	1 (0/1)	0	1 (cPKU)		NR (1)	NR (1)	-		
c. [829 T > G]; [1042C > G]	p. [Tyr277Asp]; [Leu348Val]	1 (0.38)	-	1 (1/0)	-	1 (mPKU)	0	no data	unk (0–12)	R (41)	-		
c. [829 T > G]; [1066-11G > A]	p. [Tyr277Asp]; [?]	1 (0.38)	-	-	1 (0/1)	0	1 (cPKU)		unk (0–12)	NR (0)	-		
c. [833C > A]; [1315 + 1G > A]	p. [Thr278Asn]; [?]	1 (0.38)	-	-	1 (0/1)	0	1 (cPKU)		unk	NR (0)	-		
c. [837delC]^#^; [1315 + 1G > A]	p. [His280Asnfs*61]; [?]	1 (0.38)	-	-	1 (0/1)	0	1 (cPKU)		unk	NR (0)	-		
c. [838G > A]; [838G > A]	p. [Glu280Lys]; [Glu280Lys]	11 (4.21)	-	-	11 (0/11)	0	11 (11 cPKU)		NR (1.95)	NR (1.95)	-		
c. [838G > A]; [842 + 1G > A]	p. [Glu280Lys]; [?]	1 (0.38)	-	-	1 (0/1)	0	1 (cPKU)		NR (1.95)	unk	-		
c. [838G > A]; [869A > T]^#^	p. [Glu280Lys]; [His290Leu]	1 (0.38)	-	-	1 (0/1)	0	1 (cPKU)		NR (1.95)	unk	-		
c. [838G > A]; [898G > T]	p. [Glu280Lys]; [Ala300Ser]	1 (0.38)	1 (1/0)	-	-	1 (mHP)	0	no data	NR (1.95)	R (31)	-		
c. [838G > A]; [1055delG]	p. [Glu280Lys]; [Gly352Valfs*48]	1 (0.38)	-	-	1 (0/1)	0	1 (cPKU)		NR (1.95)	unk	-		NR
c. [838G > A]; [1066-11G > A]	p. [Glu280Lys]; [?]	1 (0.38)	-	-	1 (0/1)	0	1 (cPKU)		NR (1.95)	NR (0)	-		
c. [838G > A]; [1066-3C > T]	p. [Glu280Lys]; [?]	2 (0.77)	-	-	2 (0/2)	0	2 (2 cPKU)		NR (1.95)	R	-		
c. [838G > A]; [1208C > T]	p. [Glu280Lys]; [Ala403Val]	1 (0.38)	1 (1/0)	-	-	1 (mHP)	0	no data	NR (1.95)	R (32)	-		
c. [838G > A]; [1222C > T]	p. [Glu280Lys]; [Arg408Trp]	1 (0.38)	-	-	1 (0/1)	0	1 (cPKU)		NR (1.95)	NR (1)	-		
c. [838G > A]; [1243G > A]	p. [Glu280Lys]; [Asp415Asn]	1 (0.38)	1 (1/0)	-	-	1 (mHP)	0	no data	NR (1.95)	R (93)	-		
c. [841C > T]; [1315 + 1G > A]	p. [Pro281Ser]; [?]	1 (0.38)	-	-	1 (0/1)	0	1 (cPKU)		unk	NR (0)	-		
c. [842 + 1G > A]; [1066-11G > A]	p. [?]; [?]	1 (0.38)	-	-	1 (0/1)	0	1 (cPKU)		unk	NR (0)	-		
c. [842 + 3G > C]; [842 + 3G > C]	p. [?]; [?]	2 (0.77)	-	-	2 (0/2)	0	2 (2 cPKU)		unk	unk	-		
c. [842C > T]; [842C > T]	p. [Pro281Leu]; [Pro281Leu]	2 (0.77)	-	-	2 (0/2)	0	2 (2 cPKU)		NR (1)	NR (1)	-		
c. [842C > T]; [898G > T]	p. [Pro281Leu]; [Ala300Ser]	1 (0.38)	1 (1/0)	-	-	1 (mHP)	0	3 R	NR (1)	R (31)	-		
c. [842C > T]; [926C > T]	p. [Pro281Leu]; [A309Val]	1 (0.38)	-	-	1 (0/1)	0	1 (cPKU)		NR (1)	R (44)	-		
c. [842C > T]; [1042C > G]	p. [Pro281Leu]; [Leu348Val]	2 (0.77)	-	-	2 (0/2)	0	2 (2 cPKU)		NR (1)	R (41)	-		
c. [842C > T]; [1066-11G > A]	p. [Pro281Leu]; [?]	1 (0.38)	-	-	1 (0/1)	0	1 (cPKU)		NR (1)	NR (0)	-		
c. [842C > T]; [1222C > T]	p. [Pro281Leu]; [Arg408Trp]	1 (0.38)	-	-	1 (0/1)	0	1 (cPKU)		NR (1)	NR (1)	-		
c. [842C > T]; [1241A > G]	p. [Pro281Leu]; [Tyr414Cys]	2 (0.77)	-	1 (1/0)	1 (1/0)	2 (mPKU, cPKU)	0	6 R; 1 NR	NR (1)	R^a^ (36)	-		
c. [842C > T]; [1315 + 1G > A]	p. [Pro281Leu]; [?]	3 (1.15)	-	-	3 (1/2)	1 (cPKU) minus 33 %	2 (2 cPKU)	6 cPKU NR	NR (1)	NR (0)	-		
c. [887A > G]^#^; [1222C > T]	p. [Asp296Gly]; [Arg408Trp]	1 (0.38)	-	-	1 (1/0)	1 (cPKU) minus 36 %	0	no data	unk	NR (1)	-	unclear	
c. [896 T > G]; [1222C > T]	p. [Phe299Cys]; [Arg408Trp]	1 (0.38)	-	-	1 (0/1)	0	1 (cPKU)		unk (3)	NR (1)	-		
c. [896 T > G]; [169-?_352 + ?del]	p. [Phe299Cys]; [Ex3del]	1 (0.38)	-	-	1 (0/1)	0	1 (cPKU)		unk (3)	unk	-		
c. [898G > T]; [1045 T > C]	p. [Ala300Ser]; [Ser349Pro]	1 (0.38)	-	1 (1/0)	-	1 (mPKU)	0	4 R	R (31)	NR (1)	-		
c. [898G > T]; [1066-11G > A]	p. [Ala300Ser]; [?]	1 (0.38)	-	1 (1/0)	-	1 (mPKU)	0	16 R	R (31)	NR (0)	-		
c. [898G > T]; [1315 + 1G > A]	p. [Ala300Ser]; [?]	2 (0.77)	2 (2/0)	-	-	2 (mHP)	0	3 R	R (31)	NR (0)	-		
c. [912 + 1G > A]; [1055delG]	p. [?]; [Gly352Valfs*48]	1 (0.38)	-	-	1 (0/1)	0	1 (cPKU)		unk	unk	-		NR
c. [912 + 1G > A]; [1315 + 1G > A]	p. [?]; [?]	1 (0.38)	-	-	1 (0/1)	0	1 (cPKU)		unk	NR (0)	-		
c. [912 + 2 T > C]^#^; [1042C > G]	p. [?]; [Leu348Val]	1 (0.38)	-	-	1 (0/1)	0	1 (cPKU)		unk	R (41)	-		
c. [913-8A > G]^#^; [1315 + 1G > A]	p. [?]; [?]	1 (0.38)	-	-	1 (0/1)	0	1 (cPKU)		unk	NR (0)	-		
c. [1042C > G]; [1042C > G]	p. [Leu348Val]; [Leu348Val]	1 (0.38)	-	-	1 (1/0)	1 (cPKU)	0	3 PKU R	R (41)	R (41)	-		
c. [1042C > G]; [1066-11G > A]	p. [Leu348Val]; [?]	1 (0.38)	-	-	1 (0/1)	0	1 (cPKU)		R (41)	NR (0)	-		
c. [1042C > G]; [1169A > G]	p. [Leu348Val]; [Glu390Gly]	1 (0.38)	-	1 (1/0)	-	1 (mPKU)	0	3 R	R (41)	R (72.5)	-		
c. [1042C > G]; [1222C > T]	p. [Leu348Val]; [Arg408Trp]	1 (0.38)	-	-	1 (0/1)	0	1 (cPKU)		R (41)	NR (1)	-		
c. [1045 T > C]; [1066-3C > T]	p. [Ser349Pro]; [?]	1 (0.38)	-	-	1 (0/1)	0	1 (cPKU)		NR (1)	R	-		
c. [1045 T > C]; [1162G > A]	p. [Ser349Pro]; [Val388Met]	2 (0.77)	-	-	2 (1/1)	1 (cPKU) minus 72 %	1 (cPKU) minus 20 %	2 R; 4 cPKU NR	NR (1)	R (27.5)	-		
c. [1045 T > C]; [1169A > G]	p. [Ser349Pro]; [Glu390Gly]	1 (0.38)	-	1 (1/0)	-	1 (mPKU)	0	5 R	NR (1)	R (72.5)	-		
c. [1045 T > C]; [1243G > A]	p. [Ser349Pro]; [Asp415Asn]	1 (0.38)	1 (0/1)	-	-	0	1 (mHP)		NR (1)	R (93)	-		
c. [1045 T > C]; [442-?_509 + ?del]	p. [Ser349Pro]:Ex5del]	1 (0.38)	-	-	1 (0/1)	0	1 (cPKU)		NR (1)	unk	-		
c. [1045 T > G]; [1045 T > G]	p. [Ser349Ala]; [Ser349Ala]	2 (0.77)	-	1 (1/0)	1 (0/1)	1 (mPKU) minus 71.5 %	1 (cPKU) minus 7 %	1 R	unk	unk	-	R	R
c. [1055delG]; [1055delG]	p. [Gly352Valfs*48]; [Gly352Valfs*48]	7 (2.68)	-	-	7 (0/7)	0	7 (7 cPKU)		unk	unk	-	NR	NR
c. [1055delG]; [1066-11G > A]	p. [Gly352Valfs*48]; [?]	2 (0.77)	-	-	2 (0/2)	0	2 (2 cPKU)		unk	NR (0)	-	NR	
c. [1055delG]; [1208C > T]	p. [Gly352Valfs*48]; [Ala403Val]	1 (0.38)	1 (1/0)	-	-	1 (mHP)	0	no data	unk	R (32)	-	NR	
c. [1065 + 1G > A]; [1169A > G]	p. [?]; [Glu390Gly]	1 (0.38)	1 (1/0)	-	-	1 (mHP)	0	no data	unk	R (72.5)	-		
c. [1066-2A > T]^#^; [1169A > G]	p. [Glu390Gly]; [?]	1 (0.38)	-	-	-	1 ('unk)	0	no data	unk	R (72.5)	-		
c. [1066-11G > A]; [1066-11G > A]	p. [?]; [?]	5 (1.915)	-	1 (0/1)	4 (1/3)	1 (cPKU) minus 31 %	4 (1 mPKU, 3 cPKU)	10 R; 74 NR	NR (0)	NR (0)	-		
c. [1066-11G > A]; [1162G > A]	p. [?]; [Val388Met]	1 (0.38)	-	-	1 (0/1)	0	1 (cPKU)		NR (0)	R (27.5)	-		
c. [1066-11G > A]; [1196 T > C]^#^	p. [?]; [Val399Ala]	1 (0.38)	-	-	1 (1/0)	1 (cPKU) minus 97 %	0	no data	NR (0)	unk	-		R
c. [1066-11G > A]; [1199 + 1G > C]	p. [?]; [?]	3 (1.15)	-	1 (0/1)	2 (1/1)	1 (cPKU) minus 37.5 %	2 (mPKU, cPKU)	no data	NR (0)	unk	-		unclear
c. [1066-11G > A]; [1222C > T]	p. [?]; [Arg408Trp]	1 (0.38)	-	-	1 (0/1)	0	1 (cPKU)		NR (0)	NR (1)	-		
c. [1066-11G > A]; [1223G > A]	p. [?]; [Arg408Gln]	1 (0.38)	-	-	1 (1/0)	1 (cPKU)	0	no data	NR (0)	R (49.7)	-		
c. [1066-11G > A]; [1241A > G]	p. [?]; [Tyr414Cys]	4 (1.53)	-	4 (3/1)	-	3 (mPKU)	1 (mPKU) minus 21 %	18 R; 2 NR	NR (0)	R^a^ (36)	-		
c. [1066-11G > A]; [1315 + 1G > A]	p. [?]; [?]	3 (1.15)	-	-	3 (0/3)	0	3 (3 cPKU)		NR (0)	NR (0)	-		
c. [1066-11G > A]; [510-?_706 + ?dup]^#^	p. [?]; [Ex6dup]	1 (0.38)	-	1 (0/1)	-	0	1 (mPKU)		NR (0)	unk	-		
c. [1066-11G > A]; [?]	p. [?]; [?]	1 (0.38)	-	-	1 (0/1)	0	1 (cPKU)		NR (0)	-	-		
c. [1066-3C > T]; [1180G > T]^#^	p. [?]; [Asp394Tyr]	1 (0.38)	-	1 (0/1)	-	0	1 (mPKU)		R	unk	-		
c. [1068C > G]; [1162G > A]	p. [Tyr356*]; [Val388Met]	1 (0.38)	-	-	1 (1/0)	1 (cPKU)	0	no data	NR (1)	R (27.5)	-		
c. [1139C > T]; [1180G > T]^#^	p. [Thr380Met]; [Asp394Tyr]	1 (0.38)	-	1 (1/0)	-	1 (mPKU)	0	no data	R	unk	-		
c. [1139C > T]; [1-?_60 + ?del]^#^	p. [Thr380Met]; [Ex1del]	1 (0.38)	1 (1/0)	-	-	1 (mHP)	0	no data	R	unk	-		
c. [1162G > A]; [1162G > A]	p. [Val388Met]; [Val388Met]	1 (0.38)	-	-	1 (0/1)	0	1 (cPKU)		R (27.5)	R (27.5)	-		
c. [1162G > A]; [1169A > G]	p. [Val388Met]; [Glu390Gly]	1 (0.38)	-	1 (1/0)	-	1 (mPKU)	0	8 R	R (27.5)	R (72.5)	-		
c. [1162G > A]; [1223G > A]	p. [Val388Met]; [Arg408Gln]	1 (0.38)	-	-	1 (1/0)	1 (cPKU)	0	no data	R (27.5)	R (49,7)	-		
c. [1163 T > C]^#^; [1208C > T]	p. [Val388Ala]; [Ala403Val]	1 (0.38)	-	1 (1/0)	-	1 (mPKU)	0	no data	unk	R (32)	-		
c. [1169A > G]; [1241A > G]	p. [Glu390Gly]; [Tyr414Cys]	1 (0.38)	1 (1/0)	-	-	1 (mHP)	0	no data	R (72.5)	R^a^ (36)	-		
c. [1169A > G]; [?]	p. [Glu390Gly]; [?]	1 (0.38)	1 (1/0)	-	-	1 (mHP)	0		R (72.5)	-	-		
c. [1199 + 1G > C]; [1199 + 1G > C]	p. [?]; [?]	1 (0.38)	-	-	1 (0/1)	0	1 (cPKU)		unk	unk	-		
c. [1208C > T]; [1222C > T]	p. [Ala403Val]; [Arg408Trp]	2 (0.77)	1 (1/0)	1 (1/0)	-	2 (mHP, mPKU)	0	6 R	R (32)	NR (1)	-		
c. [1208C > T]; [1315 + 1G > A]	p. [Ala403Val]; [?]	1 (0.38)	1 (1/0)	-	-	1 (mHP)	0	9 R	R (32)	NR (0)	-		
c. [1222C > T]; [1222C > T]	p. [Arg408Trp]; [Arg408Trp]	5 (1.915)	-	-	5 (0/5)	0	5 (5 cPKU)		NR (1)	NR (1)	-		
c. [1222C > T]; [1223G > A]	p. [Arg408Trp]; [Arg408Gln]	1 (0.38)	-	1 (1/0)	-	1 (mPKU)	0	no data	NR (1)	R (49.7)	-		
c. [1222C > T]; [1241A > G]	p. [Arg408Trp]; [Tyr414Cys]	5 (1.915)	-	4 (2/2)	1 (0/1)	2 (mPKU) minus 43 %, minus 66 %	3 (2 mPKU, 1cPKU)	30 R; 6 NR	NR (1)	R^a^ (36)	-		
c. [1222C > T]; [1315 + 1G > A]	p. [Arg408Trp]; [?]	3 (1.15)	-	-	3 (0/3)	0	3 (3 cPKU)		NR (1)	NR (0)	-		
c. [1222C > T]; [?]	p. [Arg408Trp]; [?]	1 (0.38)	-	1 (0/1)	-	0	1 (mPKU)		NR (1)	-	-		
c. [1223G > A]; [1241A > G]	p. [Arg408Gln]; [Tyr414Cys]	2 (0.77)	1 (1/0)	-	1 (1/0)	2 (mHP, cPKU)	0	6 R	R (49.7)	R^a^ (38,5)	-		
c. [1241A > G]; [1315 + 1G > A]	p. [Tyr414Cys]; [?]	3 (1.15)	-	1 (1/0)	2 (1/1)	2 (mPKU, cPKU) minus 34 %, minus 39 %	1 (cPKU) minus 21 %	24 PKU R; 1 PKU NR	R^a^ (36)	NR (0)	-		
c. [1241A > G]; [169-?_352 + ?del]	p. [Tyr414Cys]; [Ex3del]	1 (0.38)	-	-	1 (0/1)	0	1 (cPKU)		R^a^ (36)	unk	-		
c. [1243G > A]; [169-?_352 + ?del]	p. [Asp415Asn]; [Ex3del]	1 (0.38)	-	1 (1/0)	-	1 (mPKU)	0	no data	R (93)	unk	-		
c. [1249 T > A]; [1315 + 1G > A]	p. [Tyr417Asn]; [?]	1 (0.38)	-	1 (1/0)	-	1 (mPKU) minus 37 %	0	no data	unk	NR (0)	-	unclear	
c. [1315 + 1G > A]; [1-?_60 + ?del]^#^	p. [?]; [Ex1del]	1 (0.38)	-	-	1 (0/1)	0	1 (cPKU)		NR (0)	unk	-		
c. [?]; [?]	p. [?]; [?]	1 (0.38)	-	-	1 (0/1)	0	1 (cPKU)		-	-	-		

*Freq* frequency, *MLPA* multiplex ligation-dependent probe amplification, *mut* mutation, *NR* non responsive, *PRA* predicted residual activity, *R* responsive, *unclear* BH4-response rate comprised between 30 and 40 %, *unk* unknown, * indicates a stop codon

The most frequent genotype (*n* = 11, 4.2 %) was c. [838G > A]; [838G > A] (p.[Glu280Lys]; [Glu280Lys]). Overall, 21 genotypes had a frequency higher than 1 % (n ≥ 3). Among them, the observed phenotype was consistent with the predicted phenotype (according to the predictive residual activity) for the severe mutations only. Indeed, individuals with a combination of two mutations with low PRA (<2) were consistently associated with either mPKU or most often with cPKU. Three genotypes, c. [782G > A]; [782G > A] (p.[Arg261Gln]; [Arg261Gln], *n* = 8), c. [194 T > C]; [782G > A] (p. [Ile65Thr]; [Arg261Gln], *n* = 3) and c. [143 T > C]; [143 T > C] (p. [Leu48Ser]; [Leu48Ser], *n* = 3), were associated to severe cPKU or mPKU phenotypes despite high residual activity for both mutations (p.Arg261Gln: PRA 38.5; p.Ile65Thr: PRA 25.3; p.Leu48Ser: PRA 39). In addition, in genotypes combining a mild mutation (PRA > 20) and a severe mutation (PRA < 2), mild mutation did not always dominate. For example, c. [782G > A]; [1315 + 1G > A] (p. [Arg261Gln]; [?], *n* = 5), c. [1222C > T]; [1241A > G] (p. [Arg408Trp]; [Tyr414Cys], *n* = 5), c. [194 T > C]; [1066-11G > A] (p.[Ile65Thr]; [?], *n* = 4) and c. [782G > A]; [1066-11G > A] (p.[Arg261Gln]; [?], *n* = 4) were all associated with either cPKU or mPKU.

A total of 36.7 % of all genotypes included at least one BH4-responsive patient. 83 genotypes displayed a response rate higher than 40 % and 9 genotypes displayed a response between 30 % and 40 %. This includes 46 genotypes newly described as BH4-responsive. Among all patients, 215 displayed a genotype consisting of two alleles whose BH4-responsiveness was reported in the literature. Among those, 51 were carriers of 2 alleles previously expected to be BH4-responsive. However, only 66.7 % (34/51) responded positively to the BH4-loading test. Indeed, 100 % of mHP patients carrying 2 BH4-responsive alleles responded to BH4 administration but only 85.7 % of mPKU and 42.3 % of cPKU patients (Additional file 4: Figure S4). The presence of a single responsive mutation predicted a high rate of responsiveness in mHP (84.6 %) and mPKU (73.1 %) patients, while only 13.5 % of cPKU patients carrying one responsive mutation and one non-responsive mutation exhibited positive BH4-loading test (Additional file 4: Figure S4). Another 50 of the 215 patients whose BH4-responsiveness could be predicted according to the literature, carried two non-responsive alleles. In these patients, the observed phenotype matched the predicted phenotype in all but two (Additional file 4: Figure S4), who experienced decrease, thought moderate, in their Phe concentration (minus 31 % and minus 33 %).

Confrontation of genotypes and BH4-loading tests concluded that only patients with mHP carrying 2 responsive mutations are always BH4-responders. Response to BH4 cannot be predicted in other patients carrying either two or one responsive mutations. However, the milder the phenotype is, the most probable the BH4 test is positive.

### Associations of response to BH4-loading test with type and location of mutations

Any mutation other than missense mutation is predictive of a non-response. Indeed, first, 98.2 % of responsive patients carried at least one missense mutation. Second, frequency of BH4-responders was higher in carriers of 2 missense mutations (45.2 %) than in carriers of 1 missense mutation combined with another type of mutations – such as frameshift, nonsense or splice-site mutation (29.0 %) [Fisher exact test, see Additional file 5: Figure S5]. Third, genotypes combining frameshift, in-frame, nonsense and splice-site mutations were non-responsive, except in two cases where combinations of 2 splice site mutations gave positive - though moderate – responses (minus 31 % and 37.5 %).

We looked for a possible link between BH4-responsiveness and the localisation of mutations. The oligomerization domain seems highly relevant to responsiveness since more responders were found in carriers of one missense mutation in the oligomerization domain (71.4 %) than in carriers of one missense mutation in the catalytic (31.4 %) or regulatory (27.2 %) domains (*p* = 0.0006; Fisher exact test; [see Additional file 6: Figure S6]). In addition, even in a single domain there are differences between exons. Thus, more BH4-responders were found in carriers of missense mutations in exon 11, 12 or 8 while carriers of missense mutations in exon 7 were more often BH4-unresponsive (Fisher exact test; [Additional file 7: Figure S7]).

## Discussion

In the present study, patients were classified according to pre-treatment Phe concentration following French and European recommendations into three phenotype categories i.e. mHP (180–600 μmol/L), mPKU (600–1200 μmol/L) and cPKU (>1200 μmol/L Phe) [5, 22]. While recent European consensus publication is still pending, NIH American conference consensus, which was published at a time when our study was completed, proposed 5 categories, namely mHP not requiring treatment (<360 μmol/L), mild HP-gray zone (360–600 μmol/L), mPKU (600–900 μmol/L), moderate PKU (900–1200 μmol/L) and cPKU(>1200 μmol/L Phe) [26]. This different classification system has to be kept in mind when considering the findings and conclusions in our mHP group.

Our multicentric cohort study represents respectively 17.4 % of global cPKU and mPKU and 2.6 % of global mHP diagnosed in France since the beginning of the neonatal screening in the seventies (data from the French neonatal screening association AFDPHE, [27]). In this study, in which we included only 364 patients with both genetic testing and BH4 testing, frequency of mHP is 9 %. Frequency of mHP in all genotyped individuals (*n* = 701) in our center is 19.3 %, which is higher than the frequency in the 364 patients included in this study (9 %), but still not representative of the 39.4 % of mHP observed among the whole PKU patients screened in metropolitan France since the beginning of the neonatal screening ([27]. Underrepresentation of patients with the milder phenotypes can be partly explained by the fact that there is no recommendation for BH4-testing in patients with Phe concentration under 480 μmol/L. Several mHP are lost to follow-up, in a center dependent manner, and they miss either molecular analysis and/ or BH4 testing.

Investigation of the *PAH* gene combining dHPLC, sequencing and MLPA allowed us to reach a 99.05 % mutation detection rate, in agreement with results found in Australia: 99.0 % [28], Austria: 98.6 %, [29] or Turkey: 97.3 % [30]. dHPLC is a useful method for screening a large set of samples. It proved to be reliable in our hands and cost effective by narrowing down the number of exons needed to be sequenced. However, because of the chemistry of the method, the genetic variants may not be clearly separated. Now several factors argue that direct sequencing should be the first-line test to identify mutations in the *PAH* gene. Sequencing is carried out faster and costs have dropped down. In addition, next generation sequencing is spreading quickly and allows analyzing a large number of patients in a short time and at costs going lower and lower. Next generation sequencing also allows analyzing the whole gene including introns and regulatory regions. A spectrum of 127 mutations is reported in this study, including 82 mutations with unknown BH4-responsiveness, according to information available in the literature. This study enables us to classify 5 of them as responsive (c.434A > T, c.500A > T, c.529G > C, c.1045 T > G and c.1196 T > C). The mutation c.1196 T > C (p.Val399Ala) is positioned four nucleotides upstream the exon/intron junction in exon 11 and one base upstream the 5’ splice-site. Exon 11 is particularly vulnerable to mutations affecting splicing regulatory elements [31]. Thus, c.1197A > T and c.1199G > A have been shown to lead to exon skipping [32]. Minigene assays are needed to determine if the mutation c.1196 T > C could or not disrupt the balance between exonic splicing enhancers and silencers [31]. The c.434A > T (p.Asp145Val) mutation involves the substitution of a charged amino acid by a hydrophobic residue. The c.500A > T (p.Asn167Ile) mutation involves the substitution of a polar residue by a hydrophobic residue. The c.529G > C (p.Val177Leu) mutation leads to the substitution of a hydrophobic residue, mostly buried in the loop between C2 and C3 alpha helices [33], by a residue with similar physical and chemical properties. Another mutation affecting the same nucleotide, c.529G > A (p.Val177Met), had previously been described as BH4-responsive [10]. Residue Ser349 is an essential amino acid in the active site of the gene. The substitution of Serine349 by Alanine (c.1045 T > G) removes the hydroxyl group within hydrogen bonding distance of the iron-chelating H285 required for PAH catalytic activity. Missense mutations leading to modifications of either the protein overall hydrophobicity or the ability to form hydrogen bonds within the polypeptide backbone or between side chains may impact on the folding/conformation of PAH. Gersting and colleagues suggested that the consequences of these missense mutations might be compensated for by pharmacological doses of BH4 [7]. However, further functional studies are needed to verify that restoration of the enzymatic activity is possible by the addition of BH4 [7, 34, 35]. Another group of five mutations fitted the criteria for classification as responsive mutations but were not recognized as such. Indeed, there were observed in patients with a BH4-responsive rate comprised between 30 and 40 percent and we do believe that there responsiveness should be subsequently assessed by the long-term achievement of blood Phe target concentration under BH4 treatment.

The position and the nature of the mutation influence the activity of PAH enzyme, which in turn determines the patient phenotype [21]. Our study suggests that for any genotype to be potentially BH4-responsive it needs to contain one missense mutation. Indeed, 98.2 % of the responsive patients in this study carried at least one missense mutation. Conversely, homozygous or compound heterozygous patients carrying any other type of mutations are likely not to be BH4-responders, and administering a long term BH4-therapy to them will not yield an appropriate cost-effectiveness ratio. It is true that combinations of some splice-site mutations were reported previously to be associated with a partial BH4-responder phenotype. The explanation was that those mutations were not fully penetrant, determining a phenotype with persistent residual activity [36]. However, for these patients, based on our experience, BH4 would have rather limited effect in improving dietary Phe tolerance.

In this study, the frequency of BH4-responders reached 31.6 %. It is of note that, if the threshold of responsiveness was more severe (decrease of Phe concentration >40 %), the overall responsive rate would be 22.9 %, which is actually a more reasonable estimate of patients treated with BH4 in France (139 patients: data may 2013). Zurfluh and colleagues [10] postulated that the average frequency of patients potentially BH4-responsive in European populations is 55 %. However, these authors considered the BH4-responsive mutations to be dominant in compound heterozygous patients and classified carriers of one BH4-responsive allele as systematically responsive. This is not always true. Two mutant monomers interact to constitute the holoenzyme and there may be unexpected negative as well as positive inter-allelic complementation in compound heterozygous [4]. Indeed, we found that genotypes carrying 1 or 2 responsive mutations cannot be expected to be always BH4-responsive. Actually, only 66.7 % of patients carrying 2 responsive mutations, 35.4 % of patients carrying 1 responsive mutation and 1 non-responsive mutation and 35.2 % of patients carrying 1 responsive mutation and 1 unclassified mutation were effective BH4-responders. It must be stressed out that BH4-responsiveness depends on the disease phenotype, since 100 % of patients with mHP carrying two responsive alleles were found to be BH4-responders whereas this frequency was only 42.3 % for patients with cPKU.

Fourteen genotypes were assigned to both responsive and non-responsive patients. Similar discordances involving some of these genotypes were already described in the BioPKU database [2], as reported in Table 1. Indeed, while the c. [1066-11G > A]; [1066-11G > A] genotype, associated with a null predicted residual activity, was observed in five PKU non-responsive patients, one tPKU patient carrying this genotype showed a positive BH4 responsive test (H0 1356 μmol/L). However, this patient showed a Phe decrease limited to 31 %. A second example is c. [194 T > C]; [1222C > T] (p.[Ile65Thr]; [Arg408Trp]) genotype, associated both with one non-responsive tPKU (H0 2760 μmol/L) and one responsive tPKU (H0 1380 μmol/L) with a Phe decrease limited to 32 %. This dual BH4-responsiveness may be due to a BH4-loading test performed under a rather strict low-Phe diet with subnormal Phe concentrations. Indeed, in the case of some mutations, the stimulation effect of BH4 on residual enzymatic activity is weaker at low Phe concentrations, as previously described for c.782G > A (p.Arg261Gln) [4, 9, 34]. In our study, patients with p.[Arg261Gln]; [Arg261Gln] genotype and low Phe concentrations at the beginning of BH4-loading test were found to be BH4-unresponsive whereas patients with the same genotype but higher Phe concentrations were found to be responsive (Phe decrease of minus 66 %, minus 59 %, minus 38 %, minus 37 % and 0 % for basal Phe concentration before testing of 1080, 1236, 768, 960 and 430 μmol/L respectively). Another reason for dual responsiveness to BH4 is the intraday fluctuation during the period of the loading test [10]. This is especially true if the decrease in blood Phe concentration at 24 hour is around 30 to 40 %, like in two cPKU patients in this study. This causes to mis-conclude that the patient is BH4-responsive while his genotype is predicted to be unresponsive. However, only failure of long-term Phenylalanine dietary tolerance improvement would definitively confirm that the patient is unresponsive. A third reason is an interindividual variability in BH4 intestinal absorption or in concentration of cofactor required for PAH activity [9]. Lastly, a dual BH4-responsiveness can be due to possible variations in the protocol of the test [12, 20]. However, since the procedure was standardized (all centers used the same BH4 dose, the same time of follow-up and a common definition of responsiveness), discrepancies cannot be explained here by differences in protocols. To note, recent data from the European literature [14] showed that a 48 h-loading test would allow a better recognition of late responders . Here, a 24 h-BH4 loading test was used. Some late-responsive patients may thus have been misclassified as non-responders and some genotypes left unclassified could have been assigned a responsiveness status.

## Conclusion

This study describe the correlation between *PAH* genotyping and BH4 loading test in 364 French phenylketonuria patients. We report here that 46 genotypes and five mutations, whose BH4-responsiveness was previously unknown, are BH4-responsive. Our observations additionally suggest that mHP patients carrying two responsive alleles are likely to be always BH4-responders. Conversely, patients with 2 non-responsive alleles as well as carriers of combinations of frameshift, in-frame, nonsense, or splice-site mutations are likely to not respond to BH4. For the other cases, the presence of at least one missense responsive mutation is necessary, but not sufficient, for the patient to be responsive. In these patients, BH4-loading test and long-term monitoring of dietary Phe tolerance are the mainstay of the evaluation of BH4-responsiveness.

This study underscores the relevance of systematic incorporation of *PAH* genotyping together with BH4-loading test in the clinical evaluation of BH4-responsiveness to help distinguishing true response to BH4 administration.

## Ethics, consent and permissions

All procedures followed were in accordance with the ethical standards of the responsible committee on human experimentation and with the Helsinki Declaration of 1975. Informed consent for genotype assessment was obtained from patients (or parents) for being included in the study.

## Additional files

Additional file 1: Figure S1. Frequency of positive BH4-loading test is higher in mildest PKU phenotypes. Error bars represent the 95 % confidence intervals. Fisher exact test, ****p* ≤ 0.001, ***p* ≤ 0.01, **p* ≤ 0.05. (TIF 32 kb) Additional file 2: Figure S2. The 5 mutations identified in this study as being BH4-responsive are localized in the catalytic domain of phenylalanine hydroxylase. The PAH monomer is drawn as a ribbon representation complexed with tetrahydrobiopterin (BH4 orange balls), thienylalanine (analog substrate) and iron (red sphere). The N-terminal regulatory domain (residues 103 – 142) is in blue, the catalytic domain (residues 143–410) is in green and the dimerization motif (residues 411–427) is in purple. Mutated aminoacids are indicated in red. (TIF 1429 kb) Additional file 3: Figure S3. PAH secondary structure. Alpha-helices, beta-strands and turns across the regulatory, catalytic and oligomerization domains are indicated. Important residues are represented as well as the position of the five mutations identified in this study as being BH4-responsive. ARS: Autoregulatory sequence, CBR: Cofactor binding region. (TIF 315 kb) Additional file 4: Figure S4. Percentage of positive BH4 loading tests depending on responsiveness of both alleles in the different severity groups. The diagram shows that frequency of BH4 positive test in mHP patients carrying 2 responsive alleles is 100 %, whereas this percentage is 85.7 % in patients with mPKU and 42.3 % in patients with cPKU. A similar trend is observed for patients carrying only 1 BH4-responsive mutation. The 2 patients with tPKU and 2 non-responsive alleles classified as BH4-responders, moderately lowered the Phe concentrations (minus 31 % and minus 33 %). This decrease would be clinically irrelevant as developed in the discussion and highlights the importance of combining genotyping and BH4 test before classifying a patient as BH4 responsive or not. Fisher exact test, ****p* ≤ 0.001, ***p* ≤ 0.01, **p* ≤ 0.05. (TIF 44 kb) Additional file 5: Figure S5. Percentage of positive BH4-loading tests depending on the type of each of the two mutations of the genotypes. Missense mutations significantly correlate with positive BH4 loading test more frequently than any other type of mutation. miss: missence. ns: nonsense.fs: frameshift. del: deletion. dupl: duplication. Fisher exact test, ****p* ≤ 0.001, ***p* ≤ 0.01, **p* ≤ 0.05. (TIF 1173 kb) Additional file 6: Figure S6. Percentage of positive BH4 loading tests depending on domain localization of alleles from each genotype. Fisher exact test, ****p* ≤ 0.001, ***p* ≤ 0.01, **p* ≤ 0.05. (TIF 33 kb) Additional file 7: Figure S7. Percent of positive BH4-loading tests depending on localization of missense mutations. This figure concern only patients which carry at least one missense mutation (*n* = 311). Numbers indicated in each column represent the global number of observations (both responsive and non-responsive tests) for each column. ex: exon. Fisher exact test, *****p* ≤ 0.0001, ****p* ≤ 0.001, ***p* ≤ 0.01, **p* ≤ 0.05. (TIF 53 kb)
