# Supplementary figures and images for: Genotype-phenotype associations in French patients with phenylketonuria and importance of genotype for full assessment of tetrahydrobiopterin responsiveness
Source: Orphanet J Rare Dis. 2015 Dec 15;10:158. doi: 10.1186/s13023-015-0375-x (PMC5024853; doi:10.1186/s13023-015-0375-x)

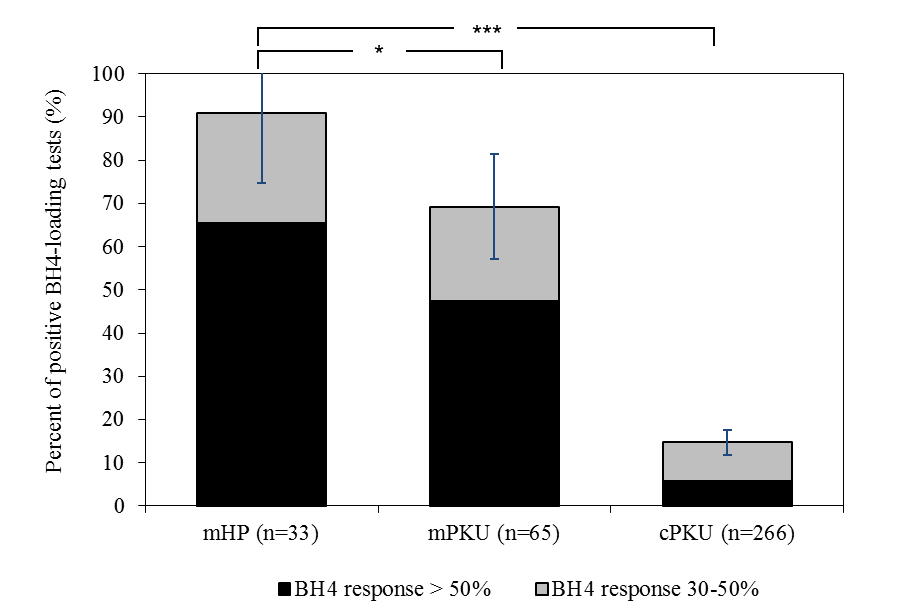

Supplement: Additional file 1: Figure S1. — Frequency of positive BH4-loading test is higher in mildest PKU phenotypes. Error bars represent the 95 % confidence intervals. Fisher exact test, ***p ≤ 0.001, **p ≤ 0.01, *p ≤ 0.05. (TIF 32 kb) [file 13023_2015_375_MOESM1_ESM.tif]

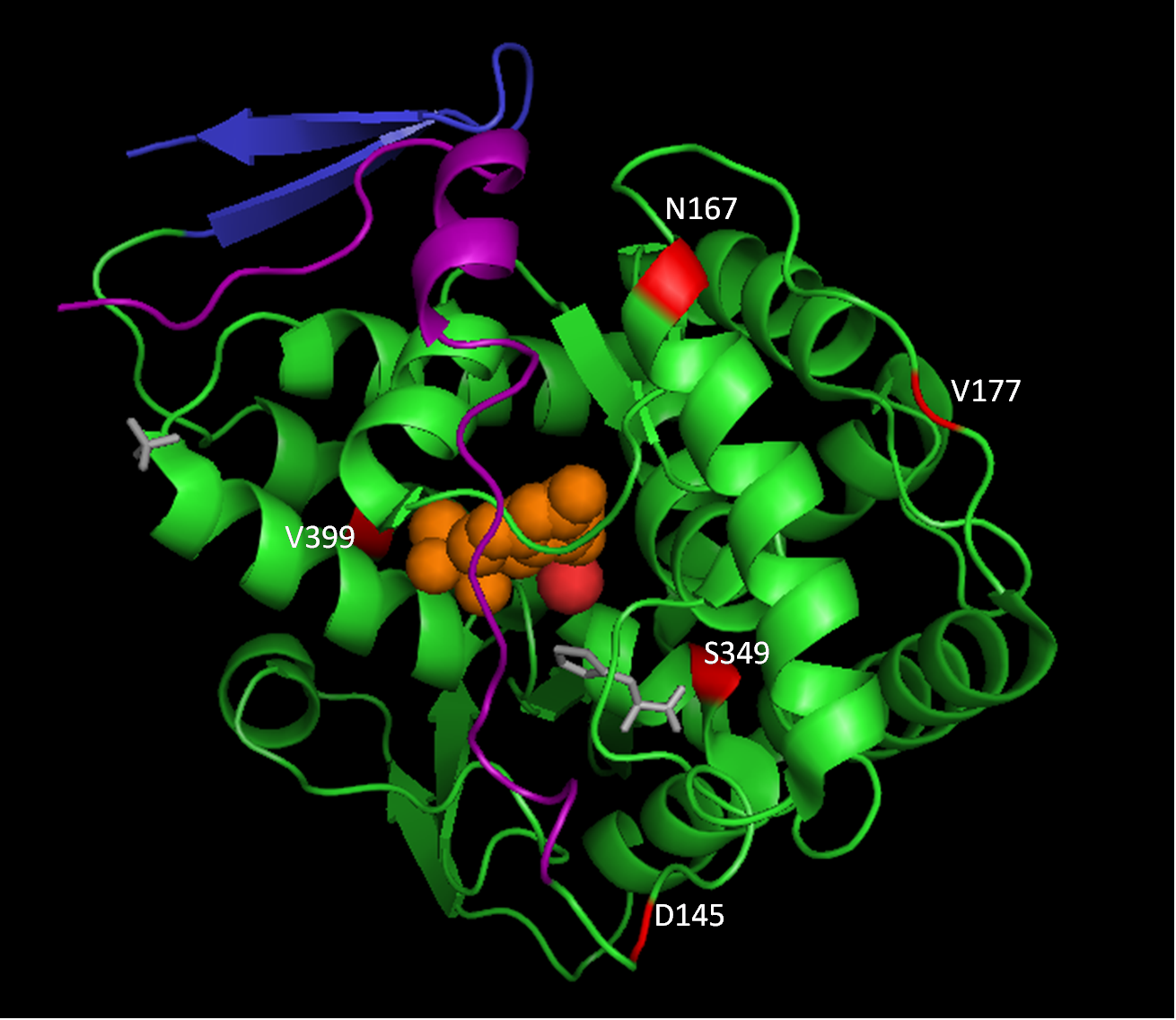

Supplement: Additional file 2: Figure S2. — The 5 mutations identified in this study as being BH4-responsive are localized in the catalytic domain of phenylalanine hydroxylase. The PAH monomer is drawn as a ribbon representation complexed with tetrahydrobiopterin (BH4 orange balls), thienylalanine (analog substrate) and iron (red sphere). The N-terminal regulatory domain (residues 103 – 142) is in blue, the catalytic domain (residues 143–410) is in green and the dimerization motif (residues 411–427) is in purple. Mutated aminoacids are indicated in red. (TIF 1429 kb) [file 13023_2015_375_MOESM2_ESM.tif]

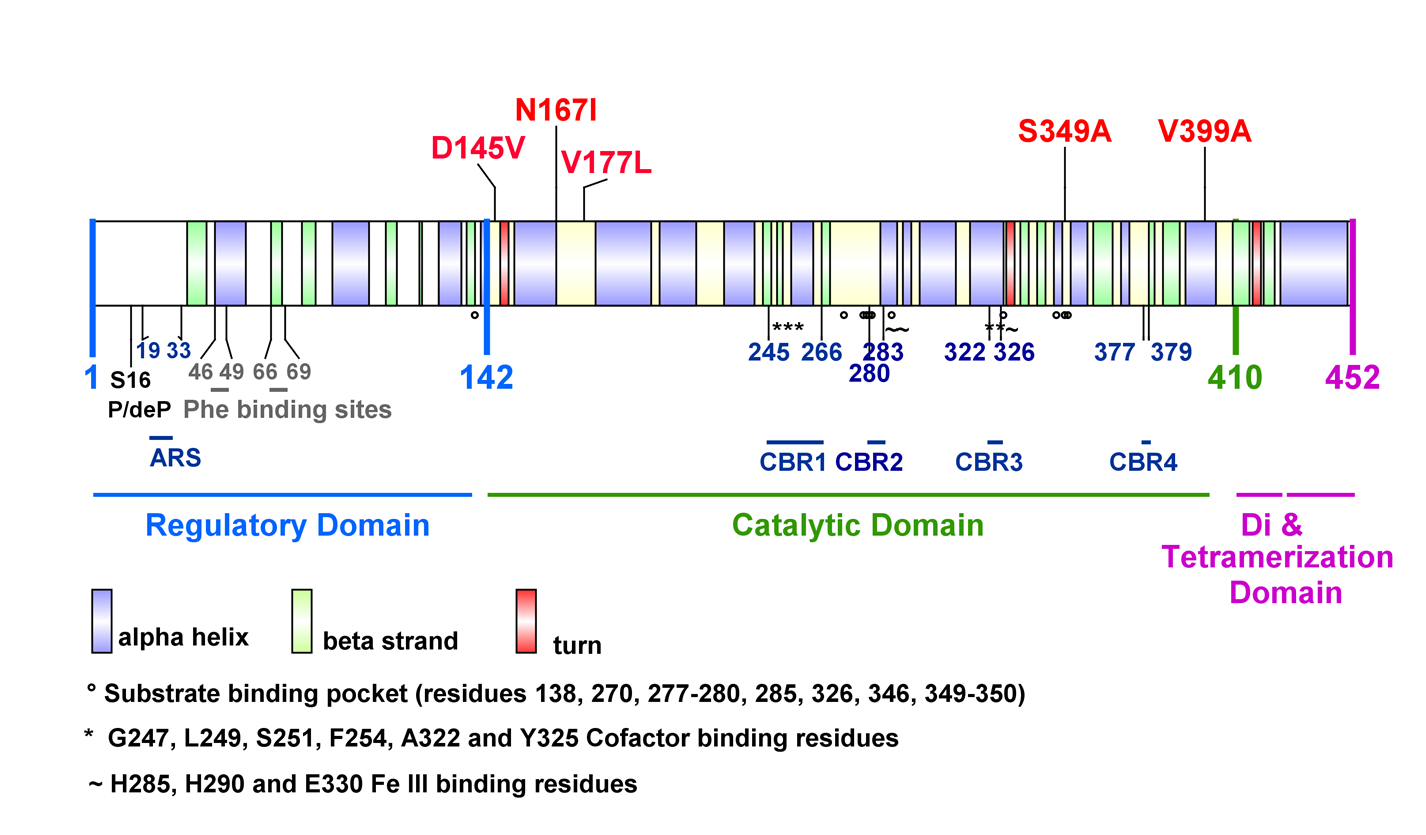

Supplement: Additional file 3: Figure S3. — PAH secondary structure. Alpha-helices, beta-strands and turns across the regulatory, catalytic and oligomerization domains are indicated. Important residues are represented as well as the position of the five mutations identified in this study as being BH4-responsive. ARS: Autoregulatory sequence, CBR: Cofactor binding region. (TIF 315 kb) [file 13023_2015_375_MOESM3_ESM.tif]

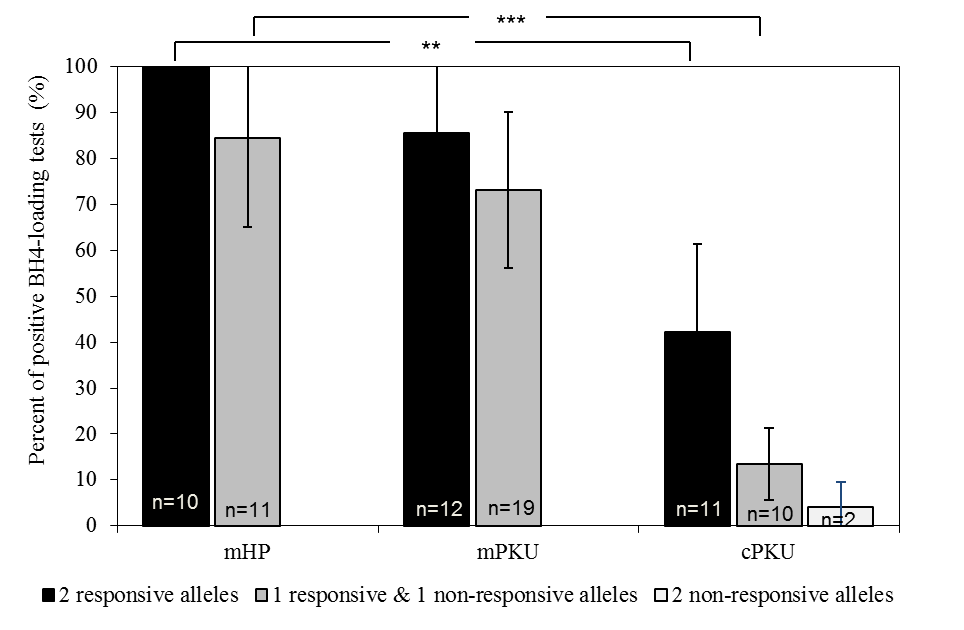

Supplement: Additional file 4: Figure S4. — Percentage of positive BH4 loading tests depending on responsiveness of both alleles in the different severity groups. The diagram shows that frequency of BH4 positive test in mHP patients carrying 2 responsive alleles is 100 %, whereas this percentage is 85.7 % in patients with mPKU and 42.3 % in patients with cPKU. A similar trend is observed for patients carrying only 1 BH4-responsive mutation. The 2 patients with tPKU and 2 non-responsive alleles classified as BH4-responders, moderately lowered the Phe concentrations (minus 31 % and minus 33 %). This decrease would be clinically irrelevant as developed in the discussion and highlights the importance of combining genotyping and BH4 test before classifying a patient as BH4 responsive or not. Fisher exact test, ***p ≤ 0.001, **p ≤ 0.01, *p ≤ 0.05. (TIF 44 kb) [file 13023_2015_375_MOESM4_ESM.tif]

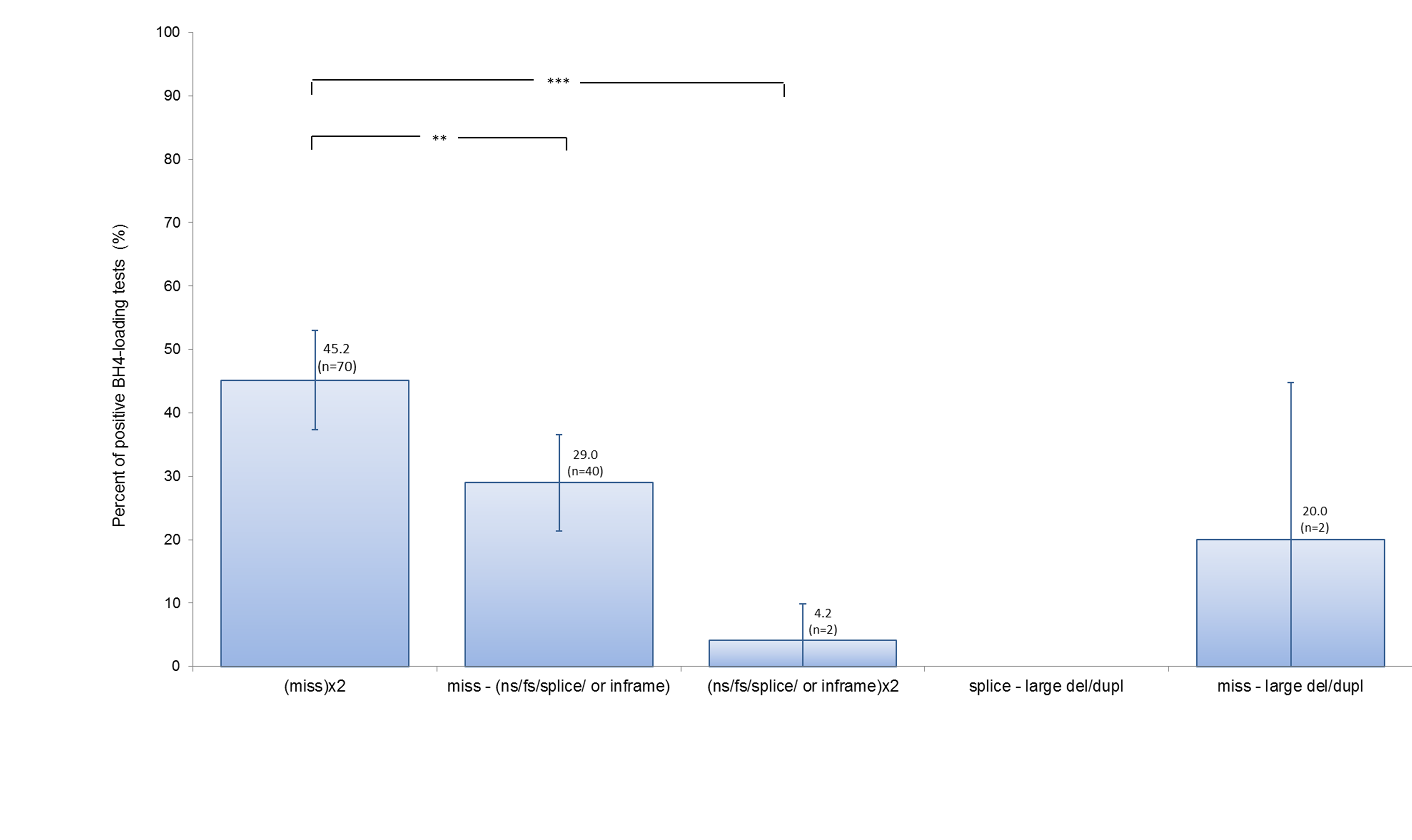

Supplement: Additional file 5: Figure S5. — Percentage of positive BH4-loading tests depending on the type of each of the two mutations of the genotypes. Missense mutations significantly correlate with positive BH4 loading test more frequently than any other type of mutation. miss: missence. ns: nonsense.fs: frameshift. del: deletion. dupl: duplication. Fisher exact test, ***p ≤ 0.001, **p ≤ 0.01, *p ≤ 0.05. (TIF 1173 kb) [file 13023_2015_375_MOESM5_ESM.tif]

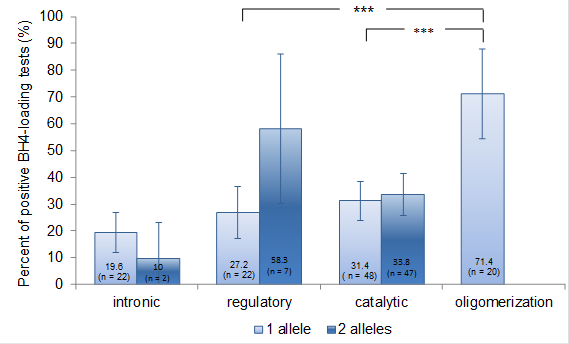

Supplement: Additional file 6: Figure S6. — Percentage of positive BH4 loading tests depending on domain localization of alleles from each genotype. Fisher exact test, ***p ≤ 0.001, **p ≤ 0.01, *p ≤ 0.05. (TIF 33 kb) [file 13023_2015_375_MOESM6_ESM.tif]

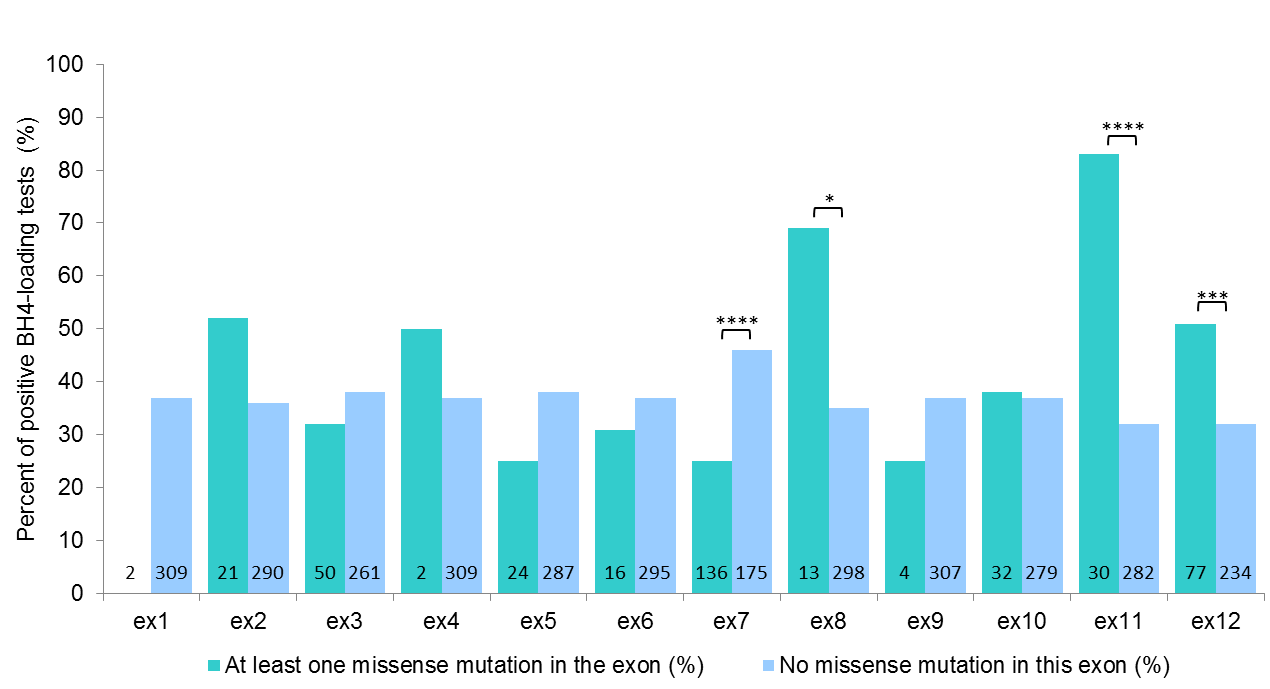

Supplement: Additional file 7: Figure S7. — Percent of positive BH4-loading tests depending on localization of missense mutations. This figure concern only patients which carry at least one missense mutation (n = 311). Numbers indicated in each column represent the global number of observations (both responsive and non-responsive tests) for each column. ex: exon. Fisher exact test, ****p ≤ 0.0001, ***p ≤ 0.001, **p ≤ 0.01, *p ≤ 0.05. (TIF 53 kb) [file 13023_2015_375_MOESM7_ESM.tif]
